# Targeting Human Protein Kinase CK2 by a Library of Indeno[1,2‐*b*]Indoles: Contribution of Thermal Shift Assay to Pre‐Screening and Co‐Crystallization to Post‐Screening

**DOI:** 10.1002/ardp.70312

**Published:** 2026-08-03

**Authors:** Matheus M. Guimarães, Christian Werner, Belen Leroy, Johana Charles, Jean Guillon, Noël Pinaud, Angélique Mularoni, Marc Jean‐Baptiste, Perrine Ximenes, Alexander Gast, Helge Prinz, Dagmar Aichele, Alan G. Gonçalves, Christelle Marminon, Zouhair Bouaziz, Joachim Jose, Jean‐Guy Delcros, Karsten Niefind, Marc Le Borgne

**Affiliations:** ^1^ Gastroenterology and Technologies for Health Team, Centre de Recherche en Cancérologie de Lyon, Centre Léon Bérard, CNRS 5286, INSERM 1052 Université Claude Bernard Lyon 1, Univ. Lyon Lyon France; ^2^ Laboratory of Synthesis of Heterocycles and Glycoconjugates, Pharmaceutical Sciences Post‐Graduation Program Federal University of Paraná Curitiba Paraná Brazil; ^3^ Institute of Biochemistry, Department of Chemistry and Biochemistry University of Cologne Koln Germany; ^4^ INSERM, CNRS, ARNA, U1212, UMR 5320, UFR des Sciences Pharmaceutiques Univ. Bordeaux Bordeaux France; ^5^ ISM‐CNRS UMR 5255 Univ. Bordeaux Talence France; ^6^ Institute of Pharmaceutical and Medicinal Chemistry, PharmaCampus University of Münster Münster Germany; ^7^ Institut des Sciences Pharmaceutiques et Biologiques (ISPB), Faculté de Pharmacie Université Claude Bernard Lyon 1, Univ. Lyon Lyon France

**Keywords:** capillary electrophoresis, indeno[1,2‐*b*]indole, protein kinase CK2, structural analysis, thermal shift assay

## Abstract

Protein kinase CK2 is the subject of numerous studies in medicinal chemistry due to its involvement in the development of several diseases, primarily cancers. Its overexpression in tumor cells is related to key processes such as tumor immune evasion and cell proliferation. The scientific approach of this study aims to investigate the thermal shift assay (TSA) as a pre‐screening tool and to complement it with a co‐crystallization approach in post‐screening. Therefore, the synthesis of seven small‐molecule CK2 inhibitors derived from indeno[1,2‐*b*]indoles was supplemented by 18 related derivatives from our in‐house compound library. The 25 molecules belong to four sub‐scaffolds, namely 4*b*,9*b*‐dihydroxy‐4*b*,5,6,7,8,9*b*‐hexahydroindeno[1,2‐*b*]indole‐9,10‐dione (D‐0), 5,6,7,8‐tetrahydroindeno[1,2‐*b*]indole‐9,10‐dione (D‐1), 9‐hydroxy‐5*H*‐indeno[1,2‐*b*]indol‐10‐one (D‐2), and 5*H*‐indeno[1,2‐*b*]indole‐6,9,10‐trione (D‐3). The most active CK2 inhibitors identified by capillary electrophoresis (CE)‐based assay belong to the D‐1 sub‐scaffold. In the TSA, these compounds also generate significant shifts of the melting temperature (Tm) of CK2, indicating a clear correlation between the results of the CE‐based assay and those of the TSA. The contribution of co‐crystallization in post‐screening also demonstrated the effectiveness of D‐1 sub‐scaffold compared with D‐0 sub‐scaffold.

## Graphical abstract

A series of 25 functionalized indeno[1,2‐*b*]indoles was selected from in‐house compound library and additional synthesis work. Capillary electrophoresis‐based and thermal shift assays, X‐ray analysis of small molecules, and co‐crystallization studies highlighted a key molecular template for the inhibitory potential of protein kinase CK2.

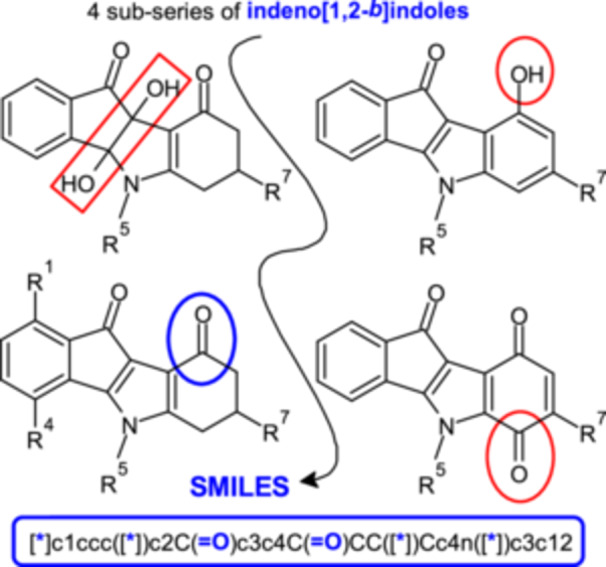

## 1 Introduction

Cancer remains one of the leading causes of death worldwide, with incidence rates expected to rise significantly in the coming decades [1]. Among various therapeutic strategies, the inhibition of protein kinases has emerged as a powerful approach to halt tumor progression and improve patient outcomes [2, 3]. As one of the largest enzyme families in humans, protein kinases are key regulators in diverse cellular processes including cell proliferation, apoptosis, transcription, and signal transduction [4]. Consequently, their dysregulation is associated with the development of many human diseases, particularly cancer [5].

Protein kinase CK2 is a highly pleiotropic serine/threonine kinase ubiquitously expressed in mammalian cells. It usually exists as a heterotetramer composed of two catalytic (α and/or α‘) and two regulatory (β) subunits. Uniquely, CK2 is constitutively active and exhibits dual co‐substrate specificity, utilizing both ATP and GTP [6]. It phosphorylates over 300 substrates involved in critical cellular pathways, many of which are linked to oncogenesis [7, 8]. High CK2 activity is associated with enhanced tumor cell survival, largely due to its anti‐apoptotic functions and its ability to promote cell proliferation [9]. This overexpression has been linked to various cancers, including leukemia and glioblastoma [10, 11]. Targeting CK2 is therefore promising not only for anticancer therapy but also for addressing other CK2‐related diseases [12], including neurodegenerative [13] and viral [14] disorders.

**Figure 1:**
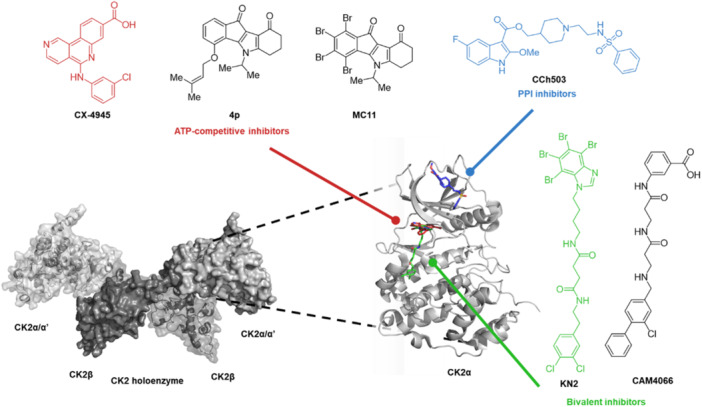
Drug design strategies applied to protein kinase CK2 holoenzyme. Structure of CK2 holoenzyme (PDB_ID 1JWH); overview of the CK2α/**CX‐4945** complex (red C‐atoms for **CX‐4945**, PDB_ID 3NGA); overview of the CK2α/**KN2** complex (green C‐atoms for **KN2**, PDB_ID 7AT5); overview of the CK2α/**CCh503** complex (blue C‐atoms for **CCh503**, PDB_ID 6FVF).

Protein kinase CK2 offers various drug design strategies [15]. CX‐4945 [16], 4p (also called D‐1‐1A5, 17), and MC11 [18] have been identified as ATP‐competitive inhibitors (Figure 1). CCh503 is a protein–protein interaction (PPI) inhibitor that specifically targets the interface between the catalytic and regulatory subunits of CK2, thereby disrupting the holoenzyme assembly [19]. In contrast, CAM4066 [20] and KN2 [21] are bivalent inhibitors, designed to simultaneously engage two distinct binding sites on the CK2α catalytic subunit, enhancing both binding affinity and selectivity. For the two bivalent inhibitors mentioned, the two interaction sites involved are the ATP‐binding site and the αD site.

**Figure 2:**
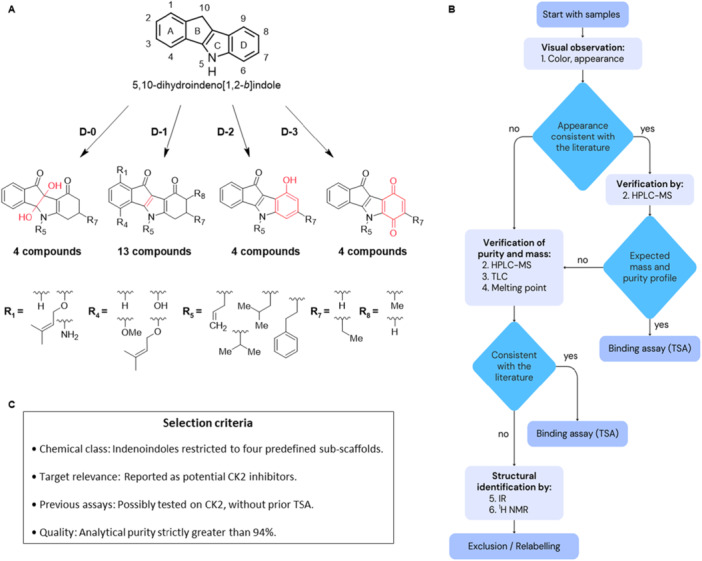
Selection of indeno[1,2‐*b*]indoles. (A) Markush formulas of the four subseries, (B) Chemical quality control and (C) Selection criteria.

The present study explores nitrogen‐containing heterocycles such as indeno[1,2‐*b*]indoles [17, 18] and related sub‐scaffolds [22] as potent inhibitors of human CK2. Of the 25 compounds studied, seven are new. Compared with previous work carried out on D‐1 (CK2) [17], D‐2 (ABCG2) [17], and D‐3 (CK2, CDC25) [22], thermal shift assay (TSA) was employed here to discriminate four sub‐scaffolds with respect to CK2 binding (Figure 2). Figure 2A illustrates the structural diversity of the indenoindole derivatives studied and classified according to four distinctive sub‐scaffolds, namely 4*b*,9*b*‐dihydroxy‐4*b*,5,6,7,8,9*b*‐hexahydroindeno[1,2‐*b*]indole‐9,10‐dione (D‐0), 5,6,7,8‐tetrahydroindeno[1,2‐*b*]indole‐9,10‐dione (D‐1), 9‐hydroxy‐5*H*‐indeno[1,2‐*b*]indol‐10‐one (D‐2) and 5*H*‐indeno[1,2‐*b*]indole‐6,9,10‐trione (D‐3) [22]. A chemical quality control flowchart (Figure 2B) shows the methodology used to ensure that each compound selected from our in‐house chemical library has the required structure and purity. Figure 2C summarizes the selection criteria used to select the indenoindole derivatives of interest. In addition, we present the chemical access to some new indenoindole derivatives, completed by crystallographic and co‐crystallographic data, and inhibition of CK2 holoenzyme for the 25 molecules studied.

## 2 Results and Discussion

### 2.1 Chemistry

**Scheme 1:**
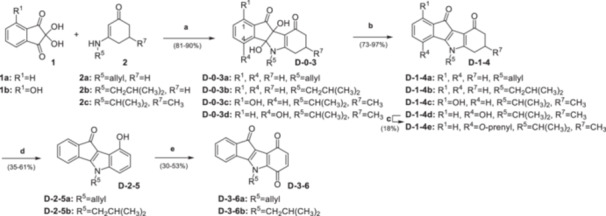
Synthesis of new indeno[1,2‐*b*]indole derivatives **D‐0‐3a**, **D‐0‐3c,d**, **D‐1‐4a**, **D‐1‐4c‐e**, **D‐2‐5a,b**, and **D‐3‐6a,b**. Reagents and conditions: (a) MeOH, r.t., 22 h; (b) (iPr_2_N)_2_SO (TIPTA) or (Et_2_N)_2_SO (TETA), DMF, AcOH, r.t., 20 h; (c) BrCH_2_CH = C(CH_3_)_2_, NaOH, DMF, r.t., 15 h; (d) 10% Pd‐C, Ph_2_O, reflux, 4 h or DDQ, dioxane, mw 140°C, 12 min.; (e) Fremy's salt, KH_2_PO_4_, acetone/water, r.t., 19 h.

**Figure 3:**
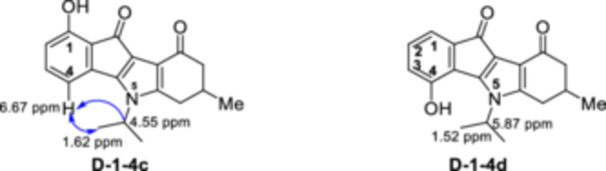
View of significant NOE interactions of 1 or 4‐hydroxylated compounds **D‐1‐4c** and **D‐1‐4d**.

Seven new indeno[1,2‐*b*]indole‐9,10‐diones **4** were synthesized in two steps (Scheme 1) according to the route previously described [17, 23, 24], that is, condensation of the corresponding ninhydrin **1** and enaminone **2**, followed by dedihydroxylation with *N,N,N’,N’*‐tetraethylthionylamide (TETA) or *N,N,N’,N’*‐tetraisopropylthionylamide (TIPTA). Compound D‐1‐4a, previously described as resulting from the rearrangement of biarylazacyclooctynone (BARAC) [25], was then obtained with an excellent yield of 87% in two steps from 3‐(2‐propen‐1‐ylamino)−2‐cyclohexen‐1‐one **2a**. As expected, the ninhydrin **1b**, with an OH in position 4, led to a mixture of the two trihydroxylated regioisomers D‐0‐3c and D‐0‐3d that were not separable by column chromatography at this stage, but fortunately were separable after the deoxygenation step [17, 26]. The structures of the two regioisomers D‐1‐4c and D‐1‐4d were assigned by NOESY experiments. Figure 3 summarizes the most significant observed correlations as blue arrows. The H aromatic at 6.67 ppm of compound D‐1‐4c correlates with the protons of the isopropyl group (CH and CH_3_), confirming the position 1 for the OH group. However, no correlation has been observed between the H aromatic protons and the protons of the isopropyl group of regioisomer D‐1‐4d, attesting the OH group at position 4. Prenylation of the poorly soluble D‐1‐4d at room temperature with 3,3‐dimethylallylbromide in the presence of NaOH afforded D‐1‐4e.

The most efficient aromatization method used for the ketonic compound D‐1‐4b to phenolic compound D‐2‐5b with 10% Pd‐C at refluxing diphenyl ether [22] was not applicable to analog D‐1‐4a as the double bond of the allyl group was also reduced. Oxidation was then conducted with 2,3‐dichloro‐5,6‐dicyano‐1,4‐benzoquinone (DDQ) under microwave irradiation at 140°C [24] to afford the expected compound D‐2‐5a with a moderate yield of 35%. Finally, the poorly soluble *para*‐quinones D‐3‐6a and D‐3‐6b were obtained with moderate yields (53% and 30%, respectively) using Fremy's salt [24].

**Figure 4:**
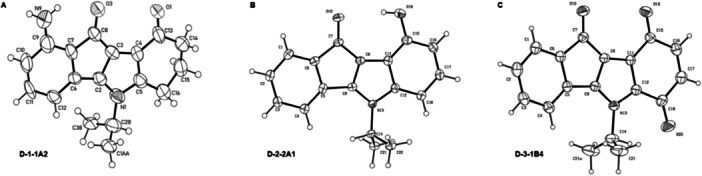
View of the crystal structures of (A) **D‐1‐1A2**, (B) **D‐2‐2A1**, and (C) **D‐3‐1B4** using our numbering system, with displacement ellipsoids drawn at a 50% probability level for **D‐1‐1A2** and 20% for **D‐2‐2A1** and **D‐3‐1B4**.

**Figure 5:**
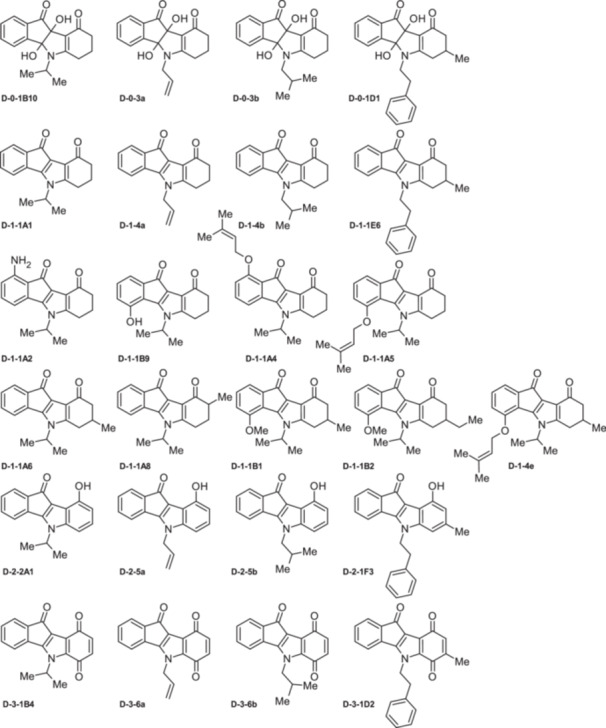
Indeno[1,2‐*b*] indoles and related sub‐scaffolds as potential CK2 inhibitors.

The 3D spatial determinations of α,β‐unsaturated ketone D‐1‐1A2, phenol D‐2‐2A1 and para‐quinone D‐3‐1B4 were established by X‐ray crystallography, and confirmed the structures in the solid state as anticipated on the basis of IR and ^1^H NMR data (Figures 4 and 5). The key bond lengths and angles of the indeno[1,2‐*b*]indol‐10‐one compounds D‐1‐1A2, D‐2‐2A1 and D‐3‐1B4 are very similar to those given in the literature for other substituted indenoindole derivatives [17, 23, 27, 28, 29, 30].

The indeno[1,2‐*b*]indol‐10‐one system of compound D‐1‐1A2, the aza‐tetracyclic moiety is almost planar with a maximum deviation found for C(15) lying 0.447(11) Å from the plane defined by the nitrogen polycyclic system. Compound D‐2‐2A1 is nearly planar with a mean out‐of‐plane deviation of 0.0271 Å with the largest deviation of 0.0542(14) Å for atom C14. Moreover, in compound D‐3‐1B4, the indeno[1,2‐*b*]indol‐10‐one moiety is almost planar with a maximum deviation from planarity of 0.2430 Å, and the maximum deviation from planarity is found for C(14) lying 0.3379(9) Å from the plane defined by the hetero‐tetracyclic system. For derivative D‐1‐1A2, these C8═O3 and C13═O double bonds are observed with lengths of 1.234(16) and 1.174(9) Å, respectively. In compound D‐2‐2A1, the C7═O10 double bond is noticed at 1.231(2) Å. In addition, the double bonds C15═O19 and C18═O20 of the 1,4‐benzoquinone moiety in D‐3‐1B4 are also confirmed by their respective lengths of 1.215(4) and 1.214(4) Å.

### 2.2 Biology

To obtain meaningful data from the TSA, we supplemented D‐0‐3a, D‐1‐4a, D‐1‐4e, D‐2‐5a, D‐2‐5b, D‐3‐6a, and D‐3‐6b with a selection of 18 indenoindoles (Figure 5) from our in‐house compound library.

Except for the four derivatives with D‐0 sub‐scaffold, other indeno[1,2‐*b*]indoles have been tested against CK2α_2_β_2_. The respective results are presented together with Log *S* data for water solubility and ΔTm (°C) values issued from the TSA method using CK2α (Table 1). Compounds with D‐1 sub‐scaffold was the series with the most significant activities. Of the 13 D‐1 compounds tested, 9 have an IC_50_ below 1 µM, including 3 with an IC_50_ below 0.050 µM, namely D‐1‐1A5 (IC_50_ **=** 0.025 μM), D‐1‐1B1 (IC_50_ **=** 0.025 μM) and D‐1‐1B2 (IC_50_ **=** 0.047 μM). Compounds D‐1‐1A5, D‐1‐1B1 and D‐1‐1B2 have in common the fact that they possess an alkoxy substituent (i.e., prenyl‐O‐ or Me‐O‐) in position 4. For compounds D‐1‐1B1 and D‐1‐1B2, an alkyl chain (Me or Et) in position 7 was added and also contributed to identifying potent inhibitors, with IC_50_ values of 0.025 µM and 0.047 µM, respectively.

**Table 1:** Inhibition of human CK2 holoenzyme, *in silico* solubility and data from the TSA by the selection of 25 indeno[1,2‐*b*]indoles.

Compound	CE‐based CK2 assay (CK2α_2_β_2_)		Water solubility	TSA (CK2α)		Reference
	Inhib. (%) ± SD at 10 μM^[^ a ^]^	IC_50_ (μM) ± SD^[^ c ^]^	Log *S* ^[^ f ^]^	Tm (°C) ± SD^[^ g ^]^	ΔTm (°C)^[^ h ^]^	
D‐0‐1B10	—	—	−2.02	43.74 ± 0.68	−0.43	[23]
D‐0‐3a	—	—	−1.86	44.16 ± 0.14	−0.01	—
D‐0‐3b	—	—	−2.57	43.91 ± 0.63	−0.26	[24]
D‐0‐1D1	—	—	−3.67	44.03 ± 0.34	−0.14	[23]
D‐1‐1A1	99	0.36	−3.14	50.28 ± 0.18	6.11	[17]
D‐1‐4a	58 ± 6	5.0 ± 0.5	−3.19	46.60 ± 0.54	2.44	—
D‐1‐4b	90 ± 2	1.5 ± 0.9	−3.73	47.72 ± 0.18	3.55	—
D‐1‐1E6	66	2.50	−4.83	44.71 ± 0.19	0.54	[17]
D‐1‐1A2	99	0.18	−3.55	51.09 ± 0.28	6.92	[31]
D‐1‐1B9	99	0.28	−3.20	50.35 ± 0.57	6.18	[17]
D‐1‐1A4	44	∼12^d^	−4.86	43.43 ± 0.68	−0.74	[17]
D‐1‐1A5	100	0.025	−4.86	50.13 ± 0.77	5.96	[17]
D‐1‐1A6	94	0.17	−3.60	48.85 ± 0.30	4.68	[17]
D‐1‐1A8	52	9.20	−3.74	45.69 ± 0.50	1.52	[17]
D‐1‐1B1	100	0.025	−3.76	50.53 ± 0.69	6.36	[32]
D‐1‐1B2	100	0.047	−4.32	49.41 ± 0.31	5.24	[32]
D‐1‐4e	97 ± 2	0.019 ± 0.005	−5.35	50.00 ± 0.98	5.83	—
D‐2‐2A1	72	2.00	−4.09	46.00 ± 0.82	1.83	[29]
D‐2‐5a	56 ± 2	5.0 ± 1.0	−4.13	44.77 ± 0.96	0.60	—
D‐2‐5b	51 ± 1	9.0 ± 1.0	−4.67	45.29 ± 0.97	1.12	—
D‐2‐1F3	42	∼12^d^	−5.70	45.96 ± 0.73	1.79	[29]
D‐3‐1B4	60	5.05	−3.22	46.52 ± 0.18	2.35	[33]
D‐3‐6a	48 ± 2	14.0 ± 6.0	−3.26	48.56 ± 0.16	4.39	—
D‐3‐6b	45 ± 2	2.8 ± 0.7	−3.80	46.24 ± 0.44	2.07	—
D‐3‐1D2	65	4.11	−4.84	43.48 ± 0.67	−0.69	[29]
CX‐4945	98 ± 4	0.0034 ± 0.0003	−5.69	56.74 ± 0.24	12.57	—
SGC‐CK2‐1	100 ± 1	0.0098 ± 0.0009	−4.71	55.09 ± 0.24	10.92	—
KDX1381	—	0.017^e^	−7.03	51.23 ± 0.31	7.06	[34]
CCh507	57^b^	45	−5.12	45.13 ± 0.32	0.96	[19]

^a^ The percentage inhibition of CK2 activity was determined for each compound at a fixed concentration of 10 μM, in triplicate. The inhibition % values without standard deviation are taken from the literature (see related references).

^b^ The inhibition % of CK2 activity was determined for compound **CCh507** at a fixed concentration of 50 μM.

^c^ For the most active compounds showing at least 45% inhibition at 10 μM, dose‐response experiments were performed to determine the IC_50_ values in triplicate.

^d^ For less potent analogs, IC_50_ values were roughly estimated based on the inhibition observed at 10 μM.

^e^ For compound **KDX1381**, IC_50_ value was determined using CK2α (see reference [34]).

^f^ Ali: topological method implemented from Ali et al. [35]; Log *S* scale: insoluble < −10 < poorly < −6 < moderately < −4 < soluble < −2 < very < 0 < highly.

^g^ For each molecule, the experiment was done in triplicate.

^h^ The ΔTm is calculated from the DMSO blank having a Tm value equal to 44.17 ± 0.42°C.

We then designed a new compound, D‐1‐4e, which can be considered a hybrid molecule of compounds D‐1‐1A5 and D‐1‐1B1, with the combination of 4‐*O*‐prenyl and 7‐Me on the same molecule. Compound D‐1‐4e is thus ranked first among our best inhibitors, with an IC_50_ equal to 0.019 µM (Table 1). Substitution at position 1 proved detrimental with the *O*‐prenyl substituent, when comparing compounds D‐1‐1A4 (IC_50_ = ∼12 µM) and D‐1‐1A5 (IC_50_ **=** 0.025 µM). Furthermore, *N*‐iPr substituent remained the best substituent compared with allyl (D‐1‐4a, IC_50_ **=** 5.0 µM) or isobutyl (D‐1‐4b, IC_50_ **=** 1.5 µM) substituents.

Derivatives with D‐2 and D‐3 sub‐scaffolds expressed lesser inhibitory potency when compared with the D‐1 sub‐scaffold. Phenolic derivatives (D‐2) did not exert very potent inhibitory activity against CK2, with IC_50_ values ranging from 2 to 12 μM. As for *para*‐quinone derivatives (D‐3‐1B4, D‐3‐6b, D‐3‐1D2), they inhibited CK2 activity by 45%–65% at 10 μM, corresponding to IC_50_ values between 2.8 and 14 μM. Moreover, the substitution of nitrogen at position 5 with bulky groups (e.g., *N*‐phenethyl for compounds D‐1‐1E6, D‐2‐1F3, and D‐3‐1D2) also appeared to have a negative effect on their inhibitory activity. In addition, the *N*‐allyl substituent does not increase activity (D‐3‐6a, inhib. % = 48, IC_50_ **=** 14 μM).

Regarding the solubility (Log *S*) [35] of the four best inhibitors (D‐1 sub‐scaffold), it is between −6 and −4 and corresponds to the “moderately soluble” scale. Their precursors *vic*‐dihydroxyindenoindoles (D‐0) are, on the other hand, more soluble, with Log *S* values between −4 and −2. Regarding the last two sub‐scaffolds, D‐2 and D‐3, their solubility is also moderately low. Nevertheless, all compounds were successfully tested by both the CE‐based CK2 assay and the TSA method without precipitation issues.

To evaluate the interaction between different potential inhibitors and a protein, the TSA method is already described with the protein kinase CK2 [36, 37]. A negative control (DMSO alone) and a known CK2 inhibitor, CX4945, were included in the study. Three compounds known to target CK2, namely SGC‐CK2‐1 (selective ATP‐competitive inhibitor) [38], KDX1381 (bivalent inhibitor) [34] and CCh507 (CK2α/CK2β interface inhibitor) [19] were also added to the study (Figure S1).

As expected, CX‐4945 induced a significant increase in the denaturation temperature (ΔTm** =** 12.57°C) compared with the DMSO control, which is in excellent agreement with the experimental data reported by Bancet et al. [37]. This result confirms the high affinity of CX‐4945 for CK2 and validates the capability of the TSA to reflect a ligand‐induced stabilization. A similar result was obtained with SGC‐CK2‐1 (ΔTm** =** 10.92°C), known to be more selective than CX‐4945. With the four best CK2 inhibitors with sub‐scaffold D‐1 (5,6,7,8‐tetrahydro[1,2‐*b*]indenoindole‐9,10‐dione) (Table 1), namely D‐1‐1A5 (IC_50_ **=** 25 nM), D1‐1B1 (IC_50_ **=** 25 nM), D‐1‐1B2 (IC_50_ **=** 47 nM) and the new one D‐1‐4e (IC_50_ **=** 19 nM), ΔTm values are among the highest observed, ranging from 5.24°C to 6.36°C. These results validate our protocol and confirm that the assay was sensitive enough to detect the thermal stabilization induced by the binding of the indenoindole‐type‐ligands to CK2α. Like the CX‐4945 and SGC‐CK2‐1, indenoindole derivatives demonstrated their ability to inhibit CK2 via an ATP‐competitive mechanism [22, 33, 39]. Furthermore, the main objective of using this TSA method was to verify how compounds belonging to other sub‐scaffolds (D‐2, D‐3) would behave, given that our previous work [22, 33, 39] had yielded inhibitory activity levels of this order: D‐1 » D‐3 and D‐2 against CK2. Taking the example of the series of compounds D‐1‐1A1, D‐3‐1B4 and D‐2‐2A1 (R^5^ **=** CH(CH_3_)_2_), the same order is also found for the ΔTm values, with 6.11°C, 2.35°C, and 1.83°C, respectively. With regard to the specific D‐0 sub‐scaffold, considered to be the simple precursor of D‐1 compounds, no SAR studies have been conducted previously. For the four D‐0 compounds tested, the observed ΔTm values are ranged from −0.43°C to −0.01°C.

**Figure 6:**
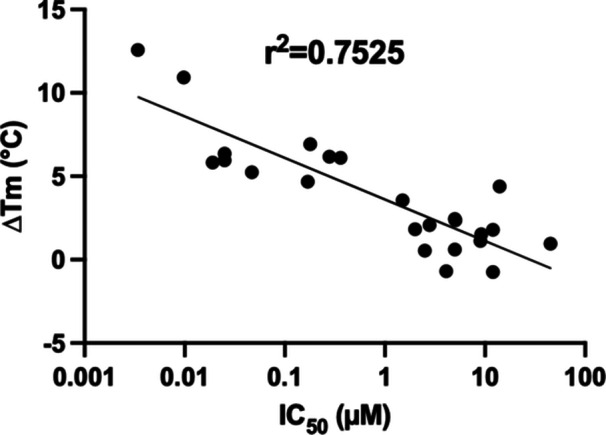
Correlation between IC_50_ (μM) and ΔTm (°C) values of 21 indeno[1,2‐*b*]indole derivatives, **CX‐4945**, **SGC‐CK2‐1** and **CCh507**.

Twenty‐five potential indenoindole inhibitors were tested and showed significant ΔTm values, particularly the D‐1 sub‐scaffold (10 compounds with a ΔTm (°C)** >** 2). Although these results did not allow direct conclusions to be drawn about the efficacy or selectivity of these compounds, they clearly indicate that they are likely to bind to CK2α and warrant further investigation. The 21 indeno[1,2‐*b*]indole‐type inhibitors were also plotted, excluding the four D‐0 compounds (no IC_50_ data available), using their IC_50_ (μM) and ΔTm (°C) values (Figure 6). For the eight D‐1 inhibitors (0.019 < IC_50_ μM < 0.36), a clear correlation is observed between IC_50_ and ΔTm values. For the other inhibitors (1.5 < IC_50_ µM < 12), this correlation is also observed.

With regard to the molecules KDX1381 and CCh503, the results are not similar. KDX1381, a known bivalent CK2 inhibitor [34], has a ΔTm of 7.06°C, which is quite similar to that of the best indenoindoles. All of these molecules interact with the ATP pocket of CK2. In contrast, for CCh503 [19], the ΔTm was measured at 0.96°C. TSA therefore appears to be less sensitive to the detection of an inhibitor at the CK2α/CK2β interface.

### 2.3 Binding Analysis of D‐0‐1B10 and D‐1‐1A1

To investigate the binding mode of D‐1‐1A1, an ATP‐competitive indenoindole‐type inhibitor [39] and its inactive precursor D‐0‐1B10, we soaked our well‐established triclinic CK2α' crystals with these compounds at high concentrations [40, 41]. Datasets were collected and solved to near‐atomic resolutions. Moreover, a control was performed by soaking D‐0‐1B10 into monoclinic CK2α' crystals. For monoclinic CK2α' crystals, no crystallization helper was added during crystallization. This approach is useful for confirming whether this compound binds to the ATP site, as very low affinity may prevent displacement of the original ligand. The data collection and refinement statistics, as well as the PDB codes, are listed in Table 2.

**Table 2:** X‐ray diffraction data and refinement statistics of published crystal structures of CK2α’ with D−0‐1B10 or D‐1‐1A1.

Complex	CK2α’, D‐1‐1A1	CK2α’, D‐0‐1B10 + 4w	CK2α’, D‐0‐1B10
PDB code	9T0U	9T2X	9TM3
*X‐ray diffraction data quality*			
Wavelength (Å)	0.97625	0.97625	0.96770
Synchrotron (beamline)	P13, EMBL/DESY	P13, EMBL/DESY	MASSIF‐3, ESRF
Space group	P1	P1	P21
Unit cell: a, b, c (Å) α, β, γ (°)	46.230, 47.415, 50.271, 113.575, 90.132, 91.171	46.274, 47.833, 50.581, 66.347, 89.803, 89.064	46.761, 72.206, 102.19290, 91.912, 90
Protomers per asymmetric unit	1	1	2
Resolution (Å) (highest resolution shell)	46.217–1.069 (1.198–1.069)	46.268–1.097 (1.279–1.097)	58.960–1.204 (1.372–1.204)
R_sym_ (%)^a^	5.6 (53.9)	5.8 (64.1)	5.7 (45.8)
CC1/2^a^	0.996 (0.520)	0.995 (0.216)	0.992 (0.619)
Signal‐to‐noise ratio (I/σ_I_)^a^	9.0 (1.8)	7.5 (1.5)	7.4 (1.5)
No. of unique reflections^a^	107,519 (5376)	80,362 (4018)	120,002 (6000)
Completeness/spherical (%)^a^	62.1 (10.7)	49.4 (6.7)	57.3 (8.9)
Completeness/ellipsoidal (%)^a^^,^ b	73.9 (23.1)	74.1 (29.0)	74.9 (30.4)
Multiplicity^a^	3.3 (3.1)	2.8 (2.5)	2.1 (1.9)
Wilson‐plot B‐factor (Å^2^)	10.79	9.58	10.45
*Structure refinement and quality*			
No. of reflections for R_work_/R_free_	107,380/4992	80,085/3850	119,991/5955
R_work_/R_free_ (%)	13.55/17.48	15.72/17.91	13.85/17.83
No. of non‐H‐atoms	3101	3195	6230
Protein	2768	2843	5621
Ligand/ion	38	46	68
Water	312	306	568
Average B‐factors (Å^2^)	19.29	16.79	17.05
Protein	17.70	15.09	15.99
Ligand/ion	19.58	41.18	24.74
Water	33.43	28.90	26.94
RMS deviations			
Bond lengths (Å)	0.008	0.009	0.011
Bond angles (°)	0.87	1.05	1.10
Ramachandran plot			
Favored (%)	97.85	96.62	96.28
Allowed (%)	2.15	3.08	3.10
Outliers (%)	0	0.31	0.62

^a^ The values in brackets refer to the highest resolution shell.

^b^ After anisotropic analysis with STARANISO.

**Figure 7:**
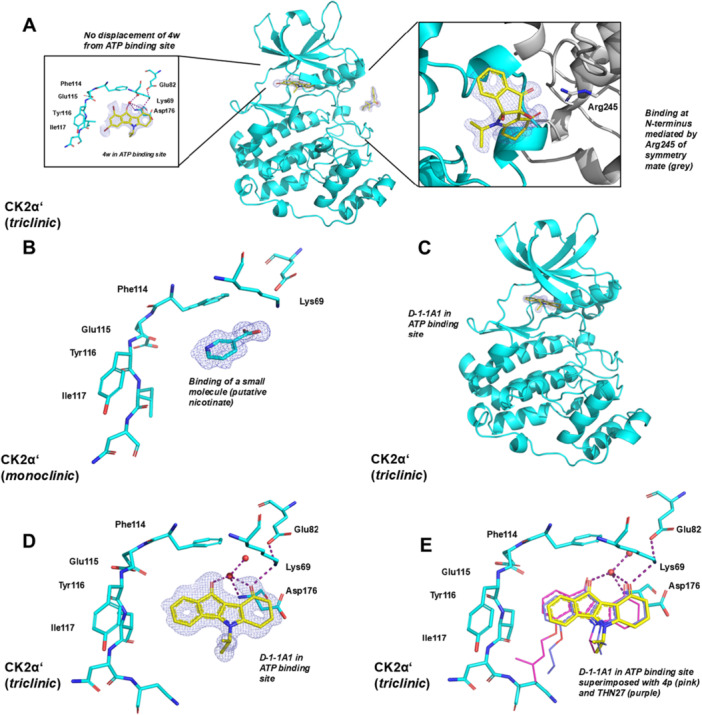
Binding analysis of **D‐0‐1B10** (A, B), and **D‐1‐1A1** (C, D, and E) to CK2α’. **D‐0‐1B10** does not bind to the ATP binding site of CK2α’ since there is no displacement of the crystallization helper molecule in the triclinic crystal system (A). However, **D‐0‐1B10** binds between two symmetry mates in the range of the N‐terminus of CK2α’ (A). The compound does neither bind to the protein in the monoclinic crystal system (B). The ATP‐binding site is empty except for a small molecule (putative nicotinate) probably derived from the expression. **D‐1‐1A1** binds to the ATP‐binding site (C and D). The binding is mediated via lysine 69, glutamate 82, and two well‐coordinated water molecules, namely the front water and the back water [40]. This binding mode is compared with the binding mode of two previously published indenoindoles [40], namely **4p** (pink, from PDB code 5OOI) and **THN27** (purple, from PDB code 6HMC), which exhibit a very similar binding mode (E).

Triclinic CK2α' crystals are grown in the presence of a crystallization helper molecule; therefore, another potential ligand must displace this compound. Here, exemplary an indenoindole itself, namely 4w [24], was used as the crystallization chaperone. This was not displaced by D‐0‐1B10, even after very extensive purging to remove 4w and soaking with D‐0‐1B10 at a very high concentration. However, we found D‐0‐1B10 bound to a putative secondary site at the N‐terminus. Anyhow, this binding is strongly mediated by two hydrogen bonds to an arginine residue of the neighboring symmetry mate, and this should therefore be considered a crystallographic artifact. The observation was confirmed in a second soaking experiment using a different crystal form of CK2α', specifically monoclinic CK2α' crystals. These crystals form without a crystallization chaperone, and only a small molecule, probably derived from the protein expression, was found in the ATP site. We modeled a putative nicotinate into the electron density. As a side note, we also found D‐0‐1B10 again at a similar crystal contact site. Also here, D‐0‐1B10 binds between the N‐terminal segment and arginine 245 of the neighboring symmetry mate. Why does D‐0‐1B10 fail to bind to the ATP site? As seen in Figure 7A, D‐0‐1B10 is strongly bent. Hence, it cannot access the ATP binding site. Overall, as expected, no binding of D‐0‐1B10 to the ATP binding site of CK2α' was detected (Figure 7A,B). These results are in good agreement with the inhibition assay and TSA.

Meanwhile, as expected, D‐1‐1A1 binds to the ATP binding site (Figure 7C,D,E). The inhibitor can easily displace the crystallization chaperone 4w after purging and soaking. The binding mode is similar to the previously published indenoindole‐type inhibitors 4p and THN27 (Figure 7E) [40]. Two highly conserved water molecules and the residues glutamate 82 and lysine 69 are involved in the binding of D‐1‐1A1 to the cosubstrate binding site. This is illustrated in Figure 7D. The dehydration step, which induces the planar shape of an indenoindole‐type inhibitor of CK2 is essential for the binding at the ATP binding site and, therefore, for the ATP‐competitive activity of this inhibitor class.

## 3 Conclusion

To further explore the indeno[1,2‐*b*]indole scaffold for the design of CK2 protein kinase inhibitors, we synthesized seven new compounds, namely D‐0‐3a, D‐1‐4a, D‐1‐4e, D‐2‐5a, D‐2‐5b, D‐3‐6a, and D‐3‐6b. D‐1 derivatives offered the best inhibition of CK2 holoenzyme (e.g., D‐1‐4e, IC_50_ **=** 0.019 μM), while D‐2 and D‐3 derivatives showed reduced inhibitory activity using a CE‐based CK2 assay.

In addition, the TSA method also proved to be a valuable pre‐screening tool to demonstrate enhanced protein stabilities arising from specific protein–ligand interactions. For example, ΔTm (°C) values for D‐1 sub‐scaffold were observed to range between 1.5 and 6.9 for 11 derivatives, with only compounds D‐1‐1A4 and D‐1‐1E6 exhibiting ΔTm (°C) values well below 0.6. This method has proven to be a fast, simple, and robust tool for pre‐screening of indenoindole‐CK2α interactions and could be systematically integrated into the early phases of our future CK2 inhibitor selection pipelines. Moreover, the TSA method has demonstrated its effectiveness in an in cellulo context, referred to as cellular TSA [42]. This approach would facilitate direct assessment of CK2 binding in cells [43]. Furthermore, co‐crystallization as a post‐screening approach proved highly valuable and, in our case, highlighted the superior effectiveness of the D‐1 sub‐scaffold (D‐1‐1A1) compared with the D‐0 sub‐scaffold (D‐0‐1B10). Overall, the combination of all these tools should optimize the discovery of new CK2 inhibitors and may be broadly applicable beyond CK2.

## 4 Experimental

### 4.1 Chemistry

#### 4.1.1 General

Chemicals are named according to IUPAC nomenclature. All of the reagents were purchased from Sigma‐Aldrich and Thermo‐Fisher Scientific. Ninhydrin 1a is commercially available, whereas 2,2,4‐trihydroxyindane‐1,3‐dione 1b was synthesized according to the literature [17]. Enaminones 2 were obtained by condensation of the corresponding cyclohexanedione with the primary amine as previously described: 3‐(2‐propen‐1‐ylamino)−2‐cyclohexen‐1‐one 2a [44], 3‐(isobutylamino)cyclohex‐2‐en‐1‐one 2b [45] and 3‐isopropylamino‐5‐methylcyclohex‐2‐en‐1‐one 2c [22].

Syntheses of the following indeno[1,2‐*b*]indole derivatives were already described: hexahydroindeno[1,2‐*b*]indole‐9,10‐diones D‐0‐1B10 [23], D‐0‐3b [24] and D‐0‐1D1 [23]; tetrahydroindeno[1,2‐*b*]indole‐9,10‐diones D‐1‐1A1, D‐1‐1E6, D‐1‐1B9, D‐1‐1A4, D‐1‐1A5, D‐1‐1A6 and D‐1‐1A8 [17], D‐1‐4b [24], D‐1‐1A2 [31], D‐1‐1B1 and D‐1‐1B2 [32]; 9‐hydroxy‐5*H*‐indeno[1,2‐*b*]indol‐10‐ones D‐2‐2A1 [29] and D‐2‐1F3 [29]; indeno[1,2‐*b*]indole‐6,9,10‐triones D‐3‐1B4 [33] and D‐3‐1D2 [29]. All of the indeno[1,2‐*b*]indole derivatives studied in this article were synthesized and characterized before the new publication, Synthesis 2026 [46], which provides additional insight into the stereochemistry of D‐0 derivatives.

Microwave reactions were conducted using a Biotage Initiator Microwave synthesizer 2.0440 W. Melting points were determined on an Electrothermal 9200 capillary apparatus. The IR spectra were recorded on a PerkinElmer Spectrum Two IR spectrometer. The ^1^H and ^13^C NMR spectra were recorded on a Bruker DRX 400 spectrometer. Chemical shifts are expressed in ppm (δ) downfield from internal TMS (tetramethylsilane) and coupling constants *J* are reported in Hertz (Hz). The following abbreviations are used: s, singlet; bs, broad singlet; d, doublet; t, triplet; q, quartet; qui, quintuplet; sept, septuplet; m, multiplet; dd, doublet of doublet; dt, doublet of triplet; Cquat, quaternary carbons. The mass spectra were performed by direct ionization (EI or CI) on a ThermoFinnigan MAT 95 XL apparatus.

Chromatographic separations were performed on silica gel columns by column chromatography (Kieselgel 300–400 mesh). All reactions were monitored by TLC on GF254 plates that were visualized under a UV lamp (254 nm). Solvent evaporation was performed under vacuum with a rotating evaporator. The purity of the final compounds was determined by uHPLC/MS on an Agilent 1290 system using an Agilent 1290 Infinity ZORBAX Eclipse Plus C18 column (2.1 mm × 50 mm, 1.8 mm particle size) or a Poroshell 120 Agilent infinity lab (2.1 mm × 50 mm, 2.7 mm particle size) with a gradient mobile phase of H_2_O/CH_3_CN (90:10, v/v) with 0.1% formic acid to H_2_O/CH_3_CN (10:90, v/v) with 0.1% formic acid at a flow rate of 0.5 mL/min, with UV monitoring at the wavelength of 254 nm with a run time of 10 min.

#### 4.1.2 Synthesis of 5‐Allyl‐4b,9b‐Dihydroxy‐4b,5,6,7,8,9b‐Hexahydroindeno[1,2‐*b*]indole‐9,10‐dione (D‐0‐3a)

An equimolar solution of ninhydrin 1a (589 mg, 3.31 mmol) and enaminone 2a (500 mg, 3.31 mmol) in 20 mL of MeOH was stirred at room temperature for 22 h. The precipitate formed was then filtered and washed with MeOH. A second quantity was obtained from the filtrate by purification by silica gel column chromatography using a mixture of dichloromethane/methanol (1:1) as eluent.

Yellow solid. 90% yield. mp: 181°C–183°C. R_*f*_ **=** 0.47 (dichloromethane/methanol, 1:1). IR (ν cm^−1^): 3358 (OH), 1722 (C**═**O), 1606 (C**═**O). ^1^H NMR (400 MHz, DMSO‐*d*_*6*_) *δ* (ppm): 7.88 (d, 1H, *J* = 7.6 Hz, H‐Ar), 7.76 (td, 1H, *J* = 7.3 Hz, *J* = 1.3 Hz, H‐Ar), 7.69 (d, 1H, *J* = 7.6 Hz, H‐Ar), 7.57 (t, 1H, *J* = 7.4 Hz, H‐Ar), 6.78 (bs, 1H, OH), 5.91‐5.82 (m, 1H, CH_2_–CH**═**CH_2_), 5.72 (bs, 1H, OH), 5.11 (dd, 1H, *J* = 9.8 Hz, *J* = 1.6 Hz, 1H of CH**═**CH_2_ cis), 5.08 (dd, 1H, *J* = 16.9 Hz, *J* = 1.6 Hz, 1H of CH**═**CH_2_ trans), 4.41–4.25 (m, 2H, NCH_2_), 2.48–2.42 (m, 1H, 1H of CH_2_−6), 2.36–2.27 (m, 1H, 1H of CH_2_−6), 2.09 (m, 2H, CH_2_−8), 2.07–2.03 (m, 1H, 1H of CH_2_−7), 1.80–1.64 (m, 1H, 1H of CH_2_−7). ^13^C NMR + DEPT (100 MHz, DMSO‐*d*_*6*_) *δ* (ppm): 197.62 (C**═**O), 188.79 (C**═**O), 165.51 (Cquat), 148.12 (Cquat), 135.40 (CH), 135.30 (CH), 134.76 (Cquat), 130.21 (CH), 125.09 (CH), 123.13 (CH), 116.79 (CH_2_), 104.88 (Cquat), 95.27 (Cquat), 83.55 (Cquat), 43.94 (CH_2_), 36.94 (CH_2_), 22.65 (CH_2_), 21.37 (CH_2_); HRMS (*m/z*): [M** +** H]^+^ calc for C_18_H_18_NO_4_, 312.1230; found 312.1228.

#### 4.1.3 Synthesis of 5‐Allyl‐5,6,7,8‐Tetrahydroindeno[1,2‐*b*]indole‐9,10‐dione (D‐1‐4a)

To a solution of 3a (500 mg, 1.60 mmol) in 4.8 mL of DMF and 0.96 mL of AcOH was added TIPTA (994 mg, 4.00 mmol). The mixture was stirred at room temperature for 22 h. The solution was then poured into 160 mL of ice and water and stirred for 1.5 h. The resulting precipitate was filtered, washed with water, and dried to give a first quantity of D‐1‐4a. The filtrate was then diluted with H_2_O and basified with NaHCO_3_. The organic layer was extracted with CH_2_Cl_2_, dried over sodium sulfate, and evaporated in vacuum to afford a second quantity of D‐1‐4a purified by silica gel column chromatography using a mixture of cyclohexane/ethyl acetate (1.5:1) as eluent.

Orange solid. 97% yield. mp 169°C. R_*f*_ = 0.10 (cyclohexane/ethyl acetate, 1.5:1). IR (ν cm^−1^): 1725 (C**═**O), 1697 (C = O). ^1^H NMR (CDCl_3_, 400 MHz) *δ* (ppm): 7.42 (dd, 1H, *J* = 6.8 Hz, *J* = 1.2 Hz, H‐Ar), 7.17 (td, 1H, *J* = 7.8 Hz, *J* = 1.2 Hz, H‐Ar), 7.09 (dd, 1H, *J* = 6.9 Hz, *J* = 1.2 Hz, H‐Ar), 6.87 (d, 1H, *J* = 7.2 Hz, H‐Ar), 6.05–5.93 (m, 1H, NCH_2_‐CH**═**CH_2_), 5.30 (dt, 1H, *J* = 10.5 Hz, *J* = 1.9 Hz, 1H of CH**═**CH_2_ cis), 5.05 (d, 1H, *J* = 17.1 Hz, *J* = 1.9 Hz, 1H of CH**═**CH_2_ trans), 4.62–4.59 (m, 2H, NCH_2_), 2.69 (t, 2H, *J* = 6.2 Hz, CH_2_−6), 2.48–2.44 (m, 2H, CH_2_−8), 2.13 (qui, 2H, *J* = 6.4 Hz, CH_2_−7). ^13^C NMR + DEPT (CDCl_3_, 100 MHz) *δ* (ppm): 192.31 (C**═**O), 184.33 (C**═**O), 152.99 (Cquat), 150.12 (Cquat), 138.89 (Cquat), 134.85 (Cquat), 132.43 (CH), 131.26 (CH), 128.50 (CH), 123.92 (CH), 120.03 (CH_2_), 118.02 (Cquat), 117.98 (Cquat), 117.28 (CH), 47.90 (CH_2_), 37.91 (CH_2_), 23.12 (CH_2_), 21.73 (CH_2_). Anal. Calcd for C_18_H_15_NO_2_ (%): C, 77.56; H, 5.38; N, 5.08. Found: C, 77.07; H, 5.35; N, 5.07.

#### 4.1.4 Synthesis of 5,6,7,8‐Tetrahydroindeno[1,2‐*b*]indole‐9,10‐diones D‐1‐4c and D‐1‐4d

A solution of 4‐hydroxyninhydrin 1b (1.00 g, 5.15 mmol) and enaminone 2c (790 mg, 5.15 mmol) in 15 mL of methanol was stirred at room temperature for 22 h. The precipitate formed was filtered and washed with methanol to get a mixture of two regioisomers 3c and 3d, which could not be separated (1.47 g, 4.30 mmol). To this mixture, dissolved in 13.5 mL of DMF, was added TETA (1.79 g, 8.61 mmol) and acetic acid (2.50 mL, 43.8 mmol). After 24 h of stirring at room temperature, crushed ice was poured, and the mixture was stirred for an additional 30 min. The precipitate formed was filtered, washed with water, and dried. A second portion was obtained by extraction of the filtrate with ethyl acetate. Regioisomers D‐1‐4c and D‐1‐4d were then separated by flash chromatography using a mixture of dichloromethane/acetone (9:1) as eluent.

1‐Hydroxy‐5‐isopropyl‐7‐methyl‐5,6,7,8‐tetrahydroindeno[1,2‐*b*]indole‐9,10‐dione (D‐1‐4c): Orange solid. 48% yield. mp > 290°C; degradation; R_*f*_ = 0.44 (dichloromethane/ethyl acetate, 9:1). IR (ν cm^−1^): 3250 (OH), 1674 (C**═**O), 1657 (C**═**O), 1616 (C**═**C). ^1^H NMR (400 MHz, CDCl_3_) *δ* (ppm): 8.94 (bs, 1H, OH), 7.10 (dd, 1H, *J* = 8.6 Hz, *J* = 7.1 Hz, H‐3), 6.67 (d, 1H, *J* = 7.1 Hz, H‐4), 6.63 (d, 1H, *J* = 8.6 Hz, H‐2), 4.55 (sept, 1H, *J* = 6.7 Hz, CHMe_2_), 2.90 (dd, 1H, *J* = 15.8 Hz, *J* = 4.0 Hz, CH_2_−6a), 2.54 (dd, 1H, *J* = 16.2 Hz, *J* = 3.2 Hz, CH_2_−8a), 2.48–2.38 (m, 2H, CH_2_−6e + H‐7), 2.20 (dd, 1H, *J* = 16.2 Hz, *J* = 11.6 Hz, CH_2_−8e), 1.62 (d, 3H, *J* = 6.8 Hz, CH_3_), 1.61 (d, 3H, *J* = 6.9 Hz, CH_3_), 1.17 (d, 3H, *J* = 6.2 Hz, CH_3_). ^13^C NMR (100 MHz, CDCl_3_) *δ* (ppm): 192.00 (C**═**O), 188.50 (C**═**O), 156.81 (Cquat), 151.05 (Cquat), 148.70 (Cquat), 135.06 (Cquat), 134.82 (CH), 120.47 (Cquat), 119.58 (CH), 119.37 (Cquat), 117.49 (Cquat), 112.63 (CH), 49.60 (NCH), 46.14 (CH_2_), 37.71 (CH_2_), 31.26 (CH), 21.96 (CH_3_), 21.81 (CH_3_), 21.40 (CH_3_). HRMS (*m/z*): [M+Na]^+^ calc for C_19_H_19_NNaO_3_, 332.1257; found: 332.1254.

4‐Hydroxy‐5‐isopropyl‐7‐methyl‐5,6,7,8‐tetrahydroindeno[1,2‐*b*]indole‐9,10‐dione (D‐1‐4d): Orange solid. 21% yield. mp > 300°C. R_*f*_ = 0.16 (dichloromethane:ethyl acetate, 9:1). IR (ν cm^−1^): 3133 (OH), 1699 (C**═**O), 1673 (C**═**O), 1599 (C**═**C). ^1^H NMR (400 MHz, DMSO‐*d*_*6*_) *δ* (ppm): 10.55 (s, 1H, OH), 7.03 (dd, 1H, *J* = 8.3 Hz, *J* = 6.9 Hz, H‐2), 6.88 (dd, 1H, *J* = 8.4 Hz, *J* = 1.0 Hz, H‐1 or H‐3), 6.84 (dd, 1H, *J* = 6.8 Hz, *J* = 1.0 Hz, H‐1 or H‐3), 5.89–5.85 (m, 1H, CHMe_2_), 3.08 (dd, 1H, *J* = 16.5 Hz, *J* = 4.8 Hz, CH_2_−6a), 2.61 (dd, 1H, *J* = 16.5 Hz, *J* = 9.6 Hz, CH_2_−6e), 2.35–2.22 (m, 2H, CH_2_−8a + H‐7), 2.16 (dd, 1H, *J* = 15.2 Hz, *J* = 11.2 Hz, CH_2_−8e), 1.52 (d, 3H, *J* = 7.1 Hz, CH_3_), 1.51 (d, 3H, *J* = 6.9 Hz, CH_3_), 1.10 (d, 3H, *J* = 6.1 Hz, CH_3_). ^13^C NMR (100 MHz, DMSO‐*d*_*6*_) *δ* (ppm): 191.01 (C**═**O), 183.22 (C**═**O), 149.23 (Cquat), 147.87 (2Cquat), 140.18 (2Cquat), 130.00 (CH), 123.38 (CH), 118.67 (2Cquat), 115.15 (CH), 50.65 (NCH), 45.90 (CH_2_), 32.37 (CH_2_), 30.99 (CH), 22.00 (CH_3_), 21.27 (CH_3_), 20.76 (CH_3_). HRMS (*m/z*): [M+Na]^+^ calc for C_19_H_19_NNaO_3_, 332.1257; found: 332.1265.

#### 4.1.5 Synthesis of 5‐Isopropyl‐7‐Methyl‐4‐[(3‐Methylbut‐2‐En‐1‐yl)Oxy]−5,6,7,8‐Tetrahydroindeno‐[1,2‐*b*]indole‐9,10‐dione (D‐1‐4e)

To a solution of indenoindole derivative D‐1‐4d (300 mg, 0.96 mmol) and sodium hydroxide (46 mg, 1.16 mmol) in dry DMF (30 mL) under an argon atmosphere was added prenyl bromide (429 mg, 2.88 mmol). After 15 h of stirring at room temperature, the DMF was evaporated, and the residue was diluted with EtOAc and water. The aqueous phase was extracted with EtOAc (4 × 25 mL). The combined organic phases were washed with water, brine, and dried over sodium sulfate. The residue was purified by column chromatography using a mixture of cyclohexane/ethyl acetate (1.5:1) as eluent.

Orange solid. 18% yield. mp 205°C (degradation). R_*f*_ = 0.25 (cyclohexane/ethyl acetate, 1.5:1). IR (ν cm^−1^): 1700 (C**═**O), 1662 (C**═**O), 1599 (C**═**C). ^1^H NMR (400 MHz, DMSO‐*d*_*6*_) *δ* (ppm): 7.18 (d, 1H, *J* = 4.8 Hz, H‐Ar), 7.17 (d, 1H, *J* = 3.2 Hz, H‐Ar), 6.96 (dd, 1H, *J* = 4.7 Hz, *J* = 3.2 Hz, H‐2), 5.83–5.79 (m, 1H, CHMe_2_), 5.51–5.47 (m, 1H, Me_2_C**═**CH), 4.66 (d, 2H, *J* = 7.0 Hz, OCH_2_), 3.08 (dd, 1H, *J* = 16.4 Hz, *J* = 4.4 Hz, CH_2_−6a), 2.62 (dd, 1H, *J* = 16.4 Hz, *J* = 9.8 Hz, CH_2_−6e), 2.35–2.22 (m, 2H, CH_2_−8a + H‐7), 2.17 (dd, 1H, *J* = 15.0 Hz, *J* = 11.0 Hz, CH_2_−8e), 1.79 (d, 3H, *J* = 1.3 Hz, Me_2_C = CH), 1.74 (d, 3H, *J* = 1.3 Hz, Me_2_C = CH), 1.47 (d, 3H, *J* = 7.0 Hz, CHMe_2_), 1.46 (d, 3H, *J* = 7.0 Hz, CHMe_2_), 1.10 (d, 3H, *J* = 6.3 Hz, CH_3_). ^13^C NMR (100 MHz, DMSO‐*d*_*6*_) *δ* (ppm): 191.01 (C**═**O), 183.07 (C**═**O), 149.58 (Cquat), 149.05 (2Cquat), 139.64 (Cquat), 139.62 (Cquat), 139.29 (Cquat), 130.40 (CH), 120.95 (Cquat), 119.97 (CH), 119.96 (Cquat), 118.62 (CH), 116.17 (CH), 65.21 (OCH_2_), 50.70 (NCH), 45.88 (CH_2_), 33.71 (CH_2_), 30.96 (CH), 25.48 (CH_3_), 21.91 (CH_3_), 21.19 (CH_3_), 20.74 (CH_3_), 17.95 (CH_3_). HRMS (*m/z*): [M+Na]^+^ calc for C_24_H_27_NNaO_3_,400.1883; found: 400.1889.

#### 4.1.6 Synthesis of 9‐hydroxy‐5*H*‐Indeno[1,2‐*b*]indol‐10‐ones 5

5‐Allyl‐9‐hydroxy‐5*H*‐indeno[1,2‐*b*]indol‐10‐one (**D‐2‐5a**): A sealed pressurized reaction vessel containing a solution of compound **D‐1‐4a** (160 mg, 0.58 mmol) and DDQ (170 mg, 0.75 mmol) in dioxane (15 mL) was irradiated for 12 min with microwave heating at 140°C. After evaporation of dioxane, 15 mL of CH_2_Cl_2_ were added, and the solution was filtered off to remove the hydroquinone. The filtrate was then concentrated and the residue purified by silica gel column chromatography using a mixture of cyclohexane:acetone (1:1) as eluent. Red solid. 35% yield. mp 178°C; R_*f*_ = 0.74 (cyclohexane/acetone, 1:1). IR (ν cm^−1^): 3332 (OH), 1667 (C**═**O). ^1^H NMR (CDCl_3_, 400 MHz) *δ* (ppm): 7.35 (d, 1H, *J* = 7.1 Hz, H‐Ar), 7.20 (td, 1H, *J* = 7.1 Hz, *J* = 1.3 Hz, H‐Ar), 7.13 (td, 1H, *J* = 7.0 Hz, *J* = 1.3 Hz, H‐Ar), 7.05 (t, 1H, *J* = 8.0 Hz, H‐Ar), 7.01 (d, 1H, *J* = 7.1 Hz, H‐Ar), 6.73 (d, 1H, *J* = 8.0 Hz, H‐Ar), 6.65 (bs, 1H, OH), 6.06–5.97 (m, 1H, CH = CH_2_), 5.28 (dt, 1H, *J* = 10.3 Hz, *J* = 1.8 Hz, 1H of CH**═**CH_2_ cis), 5.16 (dt, 1H, *J* = 17.1 Hz, *J* = 1.2 Hz, 1H of CH**═**CH_2_ trans), 4.79–4.76 (m, 2H, NCH_2_). ^13^C NMR + DEPT (CDCl_3_, 100 MHz) *δ* (ppm): 185.72 (Cquat), 156.51 (Cquat), 149.71 (Cquat), 143.51 (Cquat), 140.18 (Cquat), 135.40 (Cquat), 132.16 (CH), 131.16 (CH), 129.33 (CH), 125.30 (CH), 123.25 (CH), 118.72 (CH), 117.89 (CH_2_), 114.94 (Cquat), 112.85 (Cquat), 107.84 (CH), 103.02 (CH), 47.60 (CH_2_). HRMS (*m/z*): [M+Na]^+^ calc for C_18_H_13_NNaO_2_,298.0838; found 298.0839.

9‐Hydroxy‐5‐isobutyl‐5*H*‐indeno[1,2‐*b*]indol‐10‐one (D‐2‐5b): A solution of compound D‐1‐4b (300 mg, 1.02 mmol) in 4 mL of Ph_2_O and 0.19 g of 10% Pd/C was refluxed for 4 h. After cooling, 15 mL of MeOH were added, the solution filtered on celite and the solvent evaporated. The residue was purified by silica gel column chromatography (cyclohexane/acetone, 1:1). Red solid. 61% yield. mp 150°C. R_*f*_ = 0.79 (cyclohexane:acetone, 1:1). IR (ν cm^−1^): 3400 (OH), 1668 (C**═**O). ^1^H NMR (CDCl_3_, 400 MHz) *δ* (ppm): 7.33 (dd, 1H, *J* = 7.0 Hz, *J* = 1.2 Hz, H‐Ar), 7.21 (td, 1H, *J* = 7.0 Hz, *J* = 1.0 Hz, H‐Ar), 7.11 (t, 1H, *J* = 7.0 Hz, H‐Ar), 7.05–7.01 (m, 2H, H‐Ar), 6.72 (d, 1H, *J* = 8.2 Hz, H‐Ar), 6.64 (d, 1H, *J* = 8.1 Hz, H‐Ar), 6.44 (s, 1H, OH), 3.91 (d, 2H, *J* = 7.6 Hz, NCH_2_), 2.30–2.20 (m, 1H, NCH_2_CH), 1.00 (d, 6H, *J* = 6.8 Hz, 2CH_3_). ^13^C NMR + DEPT (CDCl_3_, 100 MHz) *δ* (ppm): 186.19 (Cquat), 157.10 (Cquat), 150.29 (Cquat), 144.42 (Cquat), 140.98 (Cquat), 136.38 (Cquat), 132.67 (CH), 129.85 (CH), 125.67 (CH), 123.76 (CH), 119.31 (CH), 115.20 (Cquat), 113.46 (Cquat), 108.21 (CH), 104.00 (CH), 53.66 (CH_2_), 30.20 (CH), 20.63 (2CH_3_). HRMS (*m/z*): [M+Na]^+^ calc for C_19_H_17_NNaO_2_, 314.1151; found 314.1143.

#### 4.1.7 General Procedure for the Synthesis of 5*H*‐Indeno[1,2‐*b*]indole‐6,9,10‐triones 6

An aqueous solution (30 mL) of Frémy's salt (4 mmol) and KH_2_PO_4_ (0.24 mmol) was added in small portions to a solution of phenol 5 (1 mmol) in acetone (30 mL). The reaction mixture was stirred at room temperature for 19 h, extracted with CH_2_Cl_2_, dried over Na_2_SO_4_, and concentrated under vacuum. The crude residue was purified by column chromatography using a mixture of cyclohexane/acetone) (1:1) as eluent.

5‐Allyl‐5*H*‐indeno[1,2‐*b*]indole‐6,9,10‐trione (D‐3‐6a): Red solid. 53% yield. mp 204°C. R_*f*_ = 0.74 (cyclohexane/acetone, 1:1). IR (ν cm^−1^): 1711 (C**═**O), 1646 (broad band, C**═**O). ^1^H NMR (CDCl_3_, 400 MHz) *δ* (ppm): 7.57 (dd, *J* = 7.3 Hz, *J* = 1.2 Hz, 1H, H‐Ar), 7.39 (td, 1H, *J* = 6.9 Hz, *J* = 1.2 Hz, H‐Ar), 7.32 (td, 1H, *J* = 6.9 Hz, *J* = 1.2 Hz, H‐Ar), 7.21 (d, 1H, J = 7.3 Hz, H‐Ar), 6.62 (d, 1H, *J* = 10.3 Hz, AB‐system, H‐7 or H‐8), 6.57 (d, 1H, *J* = 10.3 Hz, AB‐system, H‐7 or H‐8), 6.11‐6.05 (m, 1H, NCH_2_CH**═**CH_2_), 5.31 (d, 1H, *J* = 10.3 Hz, 1H of CH**═**CH_2_ cis), 5.23 (d, 2H, *J* = 5.1 Hz, NCH_2_), 5.20 (d, 1H, *J* = 17.4 Hz, 1H of CH**═**CH_2_ trans);. ^13^C NMR + DEPT (CDCl_3_, 100 MHz) *δ* (ppm): 183.47 (Cquat), 181.65 (Cquat), 178.24 (Cquat), 155.70 (Cquat), 139.90 (Cquat), 137.23 (CH), 136.42 (CH), 133.59 (CH), 133.47 (Cquat), 133.42 (Cquat), 131.15 (CH), 130.41 (CH), 124.78 (CH), 122.56 (Cquat), 120.73 (Cquat), 119.85 (CH), 118.49 (CH_2_), 49.55 (CH_2_). HRMS (*m/z*): [M+Na]^+^ calc for C_18_H_11_NNaO_3_,312.0631; found 312.0626.

5‐Isobutyl‐5*H*‐indeno[1,2‐*b*]indole‐6,9,10‐trione (D‐3‐6b): Dark red solid. 30% yield. mp 185°C. R_*f*_ = 0.75 (cyclohexane/acetone, 1:1). IR (ν cm^−1^): 1717 (C**═**O), 1651 (broad band, C**═**O). ^1^H NMR (CDCl_3_, 400 MHz) *δ* (ppm): 7.58 (d, *J* = 7.1 Hz, 1H, H‐Ar), 7.40 (t, 1H, *J* = 7.6 Hz, H‐Ar), 7.30 (d, 1H, *J* = 7.6 Hz, H‐Ar), 7.23 (t, 1H, *J* = 7.4 Hz, H‐Ar), 6.61 (d, 1H, *J* = 10.1 Hz, AB‐system, H‐7 or H‐8), 6.56 (d, 1H, *J* = 10.3 Hz, AB‐system, H‐7 or H‐8), 4.37 (d, 2H, *J* = 6.8 Hz, NCH_2_), 2.21 (m, 1H, CH(CH_3_)_2_), 1.03 (d, 6H, *J* = 6.8 Hz, 2CH_3_). ^13^C NMR + DEPT (CDCl_3_, 100 MHz) *δ* (ppm): 183.53 (Cquat), 181.74 (Cquat), 178.22 (Cquat), 155.59 (Cquat), 140.05 (Cquat), 137.41 (CH), 136.44 (CH), 133.94 (Cquat), 133.91 (Cquat), 133.51 (CH), 130.28 (CH), 124.76 (CH), 122.57 (Cquat), 120.59 (Cquat), 119.83 (CH), 54.66 (CH_2_), 30.31 (CH), 19.84 (2CH_3_). HRMS (*m/z*): [M+Na]^+^ calc for C_19_H_15_NNaO_3_, 328.0944; found 328.0938.

### 4.2 Determination of In Silico Physicochemical Properties

In our study, we used the SwissADME web tool (http://www.SwissADME.ch/) to analyze various pharmacokinetic attributes, including molecular weight, LogP, hydrogen bonding capacity, rotatable bonds, and adherence to Lipinski's guidelines [47].

### 4.3 Small Molecule X‐Ray Crystallography

The structure of compounds D‐1‐1A2, D‐2‐2A1, and D‐3‐1B4 has been established by X‐ray crystallography. Orange‐red single crystal (0.20 c 0.20 × 0.20 mm^3^) of D‐1‐1A2 was obtained after 7 days at 21°C from a closed methanol:chloroform (30:70) solution heated at 50°C during 5 min followed by instantaneous cooling at –20°C during 30 s: monoclinic, space group P2_1_/c11, *a* = 10.7615(11) Å, *b* = 14.9256(15) Å, *c* = 18.7476(16) Å, α = 90°, β = 90°, γ = 90°, *V* = 3011.3(5) Å^3^, Z = 4, *δ* (calcd) = 0.649 Mg.m^3^, FW = 294.34 for C_18_H_18_N_2_O_2_, *F*(000) = 624. Crystallographic data were acquired at CESAMO (UMR 5255) on a Bruker APEX 2 DUO. Red single crystal (0.12 × 0.12 × 0.02 mm^3^) of D‐2‐2A1 was obtained after 9 days at 21°C from a closed ethanol:chloroform (30:70) solution preliminary heated at 50°C during 5 min followed by instantaneous cooling at –20°C during 30 s: monoclinic, space group P21/n, *a* = 11.2637(11) Å, *b* = 6.8066(6) Å, *c* = 17.8741(17) Å, α = 90°, β = 101.157(7)°, γ = 90°, *V* = 1344.5(2) Å^3^, Z = 4, *δ*(calcd) = 1.370 Mg.m^–3^, FW = 277.31 for C_18_H_15_NO_2_, *F*(000) = 352. Red single crystal (0.09 × 0.02 × 0.02 mm^3^) of D‐3‐1B4 was obtained after 5 days at 21°C from a closed methanol:chloroform (25:75) solution heated at 50°C during 5 min followed by instantaneous cooling at –20°C during 30 s: orthorhombic, space group Pnma, *a* = 10.1081(9) Å, *b* = 77.2207(8) Å, *c* = 19.1673(18) Å, α = 90°, β = 90°, γ = 90°, *V* = 1399(2) Å^3^, Z = 4, *δ*(calcd) = 1.383 Mg.m^–3^, FW = 291.29 for C_18_H_13_NO_3_, *F*(000) = 352.

Full crystallographic results have been deposited at the Cambridge Crystallographic Data Centre (CCDC‐1544103, CCDC‐832080, CCDC‐832081), UK, as Supporting Information [48]. The data were corrected for Lorentz and polarization effects and for empirical absorption correction [49]. The structure was solved by direct methods Shelx 97 [50] and refined using Shelx 97 [51] suite of programs.

### 4.4 Capillary Electrophoresis‐Based Assay

CK2 activity was determined by a capillary electrophoresis assay as previously reported [52] with modifications. For CK2‐alpha, a C‐terminally truncated variant (aa 1‐335) with an N‐terminal (His)_6_‐tag was used. During purification, the C‐terminal part is known to be cleaved off, which has no impact on enzymatic activity [53]. CK2‐beta was used as a C‐terminally truncated variant (aa 1‐193), with an N‐terminal (His)_6_‐tag. This truncated variant has been described to be more stable and have a lower tendency to form aggregates without functional constraint [54]. Both CK2‐alpha and CK2‐beta are of human origin.

Briefly, 98 μL kinase buffer (150 mM NaCl, 25 mM MgCl_2_, 25 mM Tris/HCl, pH 7.5) containing 0.25 μg CK2α_2_β_2_ was supplemented with 2 μL of the test compound dissolved in DMSO. After incubation for 10 min at 37°C, the kinase reaction was initiated by adding 100 μL assay buffer (150 mM NaCl, 25 mM MgCl_2_, 228 μM substrate peptide RRRDDDSDDD, 120 μM ATP, 25 mM Tris/HCl, pH 7.5). After 15 min at 37°C, the reaction was stopped by adding 25 μL EDTA (0.5 M, pH 8.0). For the determination of the IC_50_ value, dose‐dependent activity determinations were performed with test compound concentrations ranging from 1 nM to 10 μM. A control without test compound, but the same volume of DMSO corresponded to 100% activity. Each data point was the result of three independent measurements. IC_50_ values were calculated using GraphPad Prism 5 (La Jolla, CA, USA).

### 4.5 Thermal Shift Assay

The TSA was performed on a LightCycler 480 Real‐Time PCR System (Roche) in 96‐well white plates (Armadillo plate, Thermo Scientific) using an integration time of 120 ms. Each well contained 10 μL of PBS‐0.9% glycerol‐5% DMSO containing 5 μg of full‐length human CK2α, purified as described previously [55], 2.5× SYPRO Orange (Life Technologies) and 10 µM ligand (indeno[1,2‐*b*]indole derivative, CX‐4945, SGC‐CK2‐1, KDX1381 or CCh507). All assays were carried out in triplicate. Each plate was sealed with an optically clear foil and centrifuged for 1 min at 300 rpm before performing the assay. The plates were heated from 20°C to 80°C at a heating rate 0.01°C/s. The fluorescence intensity was recorded at a rate of 50 acquisitions per °C with excitation at 483 nm and emission at 568 nm.

Melting temperatures (Tm, °C) were determined using the TSA‐CRAFT software that enables automatic analysis of TSA data exported from the Roche Lightcycler 480 software [56].

### 4.6 Structural Analysis of the Binding Mode of D‐0‐1B10 and D‐1‐1A1 to Ck2α'

Triclinic CK2α' crystals with the soluble mutant CK2α'^C336S^ allow studying the binding mode of indeno[1,2‐*b*]indoles at near‐atomic resolution [40, 41]. The protein for this was expressed as described earlier [57]. CK2α' was crystallized using a previously published protocol [41]. In this work, we varied the crystallization helper inhibitor to compound 4w [24].

Crystals were extensively purged from unbound compound. Complex formation was achieved by soaking at a very high concentration (calculated to be 100 mM in the drop) of compound. Crystals were cryo‐protected by soaking in 30% ethylene glycol solved in mother liquor. Crystals were flash‐frozen in liquid nitrogen.

Data collections were carried at 100 °K. Data were processed using the automated autoPROC pipeline [58], which uses XDS [59], Pointless and Aimless [60] from the ccp4i suite [61] and performs anisotropy analysis with Staraniso [62]. Phasing was done my molecular replacement in PHASER [63] using 6HMQ [40] as reference structure. Model building and refinement cycles were conducted in AutoBuild [64], Coot [65], and Phenix [66]. D‐1‐1A1 was parametrized in eLBOW [67], D‐0‐1B10 was parametrized in Grade2 [68].

As a control, crystals of a different crystal form (monoclinic crystal system) of CK2α' were soaked with D‐0‐1B10. These crystals grow without an additional crystallization chaperone [41]. A total of 3 µL of 5 mg/mL protein was mixed with 3 µL of a slightly altered reservoir solution containing 810 mM LiCl, 250 mM Tris‐HCl, and 28% (w/v) PEG6000. Crystals were optimized by microseeding and macroseeding. The soaking and cryo‐protection procedures were performed as described above. Data were processed as described above.

Data were collected on beamline MASSIF‐3 [69] of the ESRF [70] and P13 (PETRA III) of the EMBL outstation in Hamburg [71].

## Conflicts of Interest

The authors declare no conflicts of interest.

## Supporting information


Supporting File 1

Supporting File 2

## Data Availability

The CK2α'/**D‐1‐1A1** complex structure is published in the PDB under the accession code 9T0U, the CK2α'/**D‐0‐1B10** + **4w** complex structure under the accession code 9T2X and the monoclinic CK2α'/**D‐0‐1B10** complex under the accession code 9TM3, with the dataset linked under doi: 10.15151/esrf‐dc‐2423437086. Other data are available on request.
