# Supplementary material for: Targeting Human Protein Kinase CK2 by a Library of Indeno[1,2‐b]Indoles: Contribution of Thermal Shift Assay to Pre‐Screening and Co‐Crystallization to Post‐Screening
Source: Arch Pharm (Weinheim). 2026 Aug 3;359(8):e70312. doi: 10.1002/ardp.70312 (PMC13430583; doi:10.1002/ardp.70312)
Supplement: Supplementary file 1 — Supporting File 1 [file ARDP-359-e70312-s002.docx]

**Supplementary Material**

Targeting Human Protein Kinase CK2 by a Library of Indeno[1,2-*b*]indoles: Contribution of Thermal Shift Assay to Pre-screening and Co-crystallization to Post-screening

Matheus M. Guimarães^1,2^, Christian Werner^3^, Belen Leroy^1^, Johana Charles^1^, Jean Guillon^4^, Noël Pinaud^5^, Angélique Mularoni^1^, Marc Jean-Baptiste^1^, Perrine Ximenes^1^, Alexander Gast^6^, Helge Prinz^6^, Ema Vérel^1^, Dagmar Aichele^6^, Alan G. Gonçalves^2^, Christelle Marminon^1^, Zouhair Bouaziz^7^, Joachim Jose^6^, Jean-Guy Delcros^1^, Karsten Niefind^3^, Marc Le Borgne^1,^*

1 Gastroenterology and Technologies for Health Team, Centre de Recherche en Cancérologie de Lyon, Centre Léon Bérard, CNRS 5286, INSERM 1052, Université Claude Bernard Lyon 1, Univ. Lyon, Lyon, France

2 Laboratory of Synthesis of Heterocycles and Glycoconjugates, Pharmaceutical Sciences Post-Graduation Program, Federal University of Paraná, Curitiba, PR, Brazil

3 Institute of Biochemistry, Department of Chemistry and Biochemistry, University of Cologne, Koln, Germany

4 INSERM, CNRS, ARNA, U1212, UMR 5320, UFR des Sciences Pharmaceutiques, Univ. Bordeaux, Bordeaux, France

5 ISM-CNRS UMR 5255, Univ. Bordeaux, Talence, France

6 Institute of Pharmaceutical and Medicinal Chemistry, PharmaCampus, University of Münster, Münster, Germany

7 Institut des Sciences Pharmaceutiques et Biologiques (ISPB), Faculté de Pharmacie, Université Claude Bernard Lyon 1, Univ. Lyon, Lyon, France

*Correspondence:

Prof. Dr., Marc Le Borgne, Gastroenterology and technologies for health, Cancer Research Center of Lyon, Faculty of Pharmacy-ISPB, 8 avenue Rockefeller, F-69373 Lyon Cedex 8, France

Email: marc.le-borgne@univ-lyon1.fr

***Contents***

Figure S1 Page 2

Chemistry spectral data

1H and 13C NMR, and HRMS for 4b,9b-dihydroxy-4b,5,6,7,8,9b-hexahydroindeno[1,2-*b*]indole-9,10-dione (**D-0-3a**) Page 3

1H and 13C NMR, and HRMS for 5,6,7,8-tetrahydroindeno[1,2-*b*]indole-9,10-diones (**D-1-4a**, **D-1-4c**, **D-1-4d**, **D-1-4e**) Page 7

1H and 13C NMR, and HRMS for 9-hydroxy-5*H*-indeno[1,2-b]indol-10-ones (**D-2-5a**, **D-2-5b**) Page 25

1H and 13C NMR, and HRMS for 5*H*-indeno[1,2-*b*]indole-6,9,10-triones (**D-3-6a**, **D-3-6b**) Page 33

**FIGURE S1** Structures of **SGC-CK2-1**, **KDX1381** and **CCh507**.

D-0-3a purity


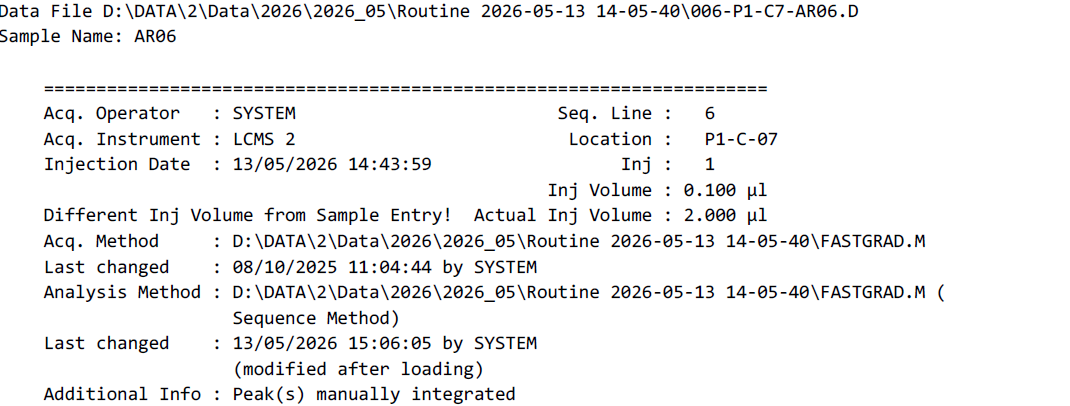


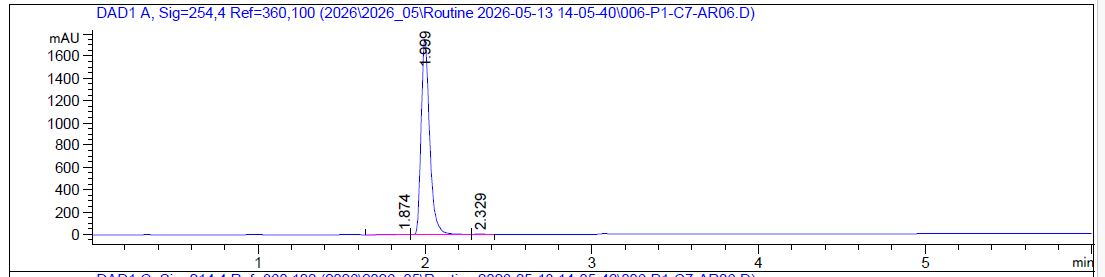


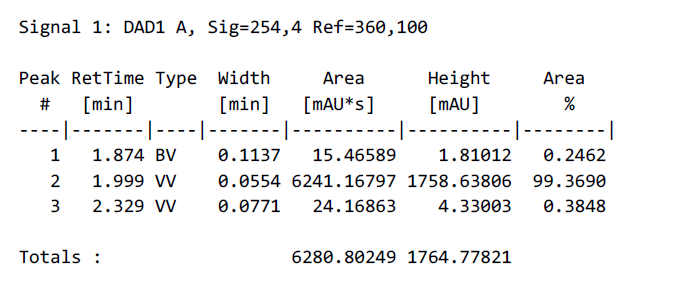


D-0-3a HRMS

D-1-4a purity


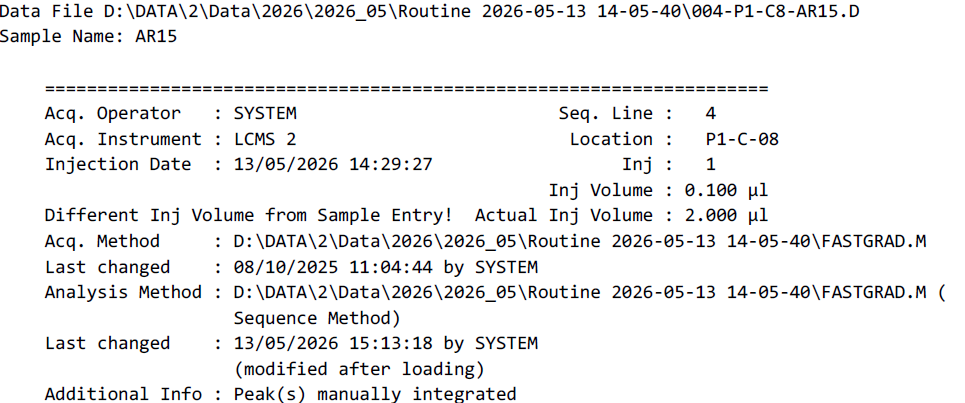


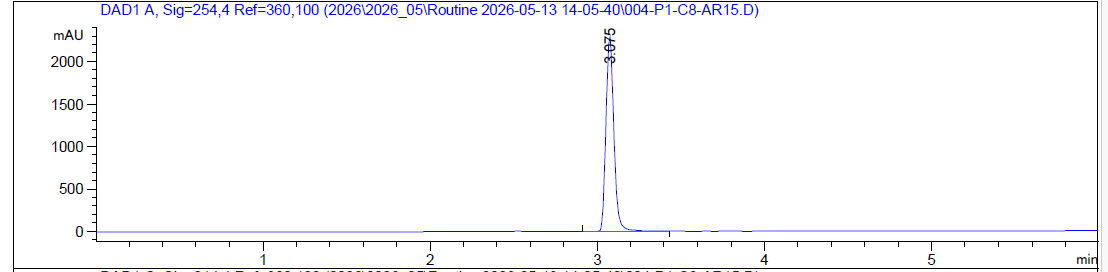


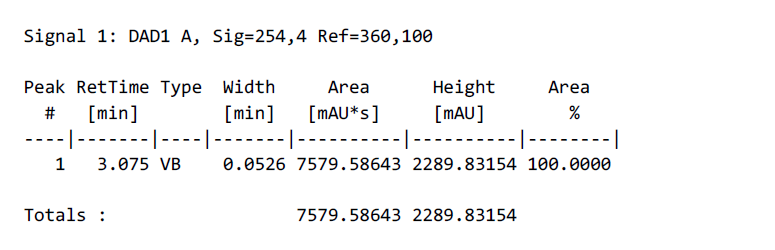


D-1-4a elemental analysis

D-1-4c- NOESY DMSO

D-1-4c purity – intermediate

D-1-4c HRMS

D-1-4d- NOESY DMSO

D-1-4d purity – intermediate for access to D-1-4e


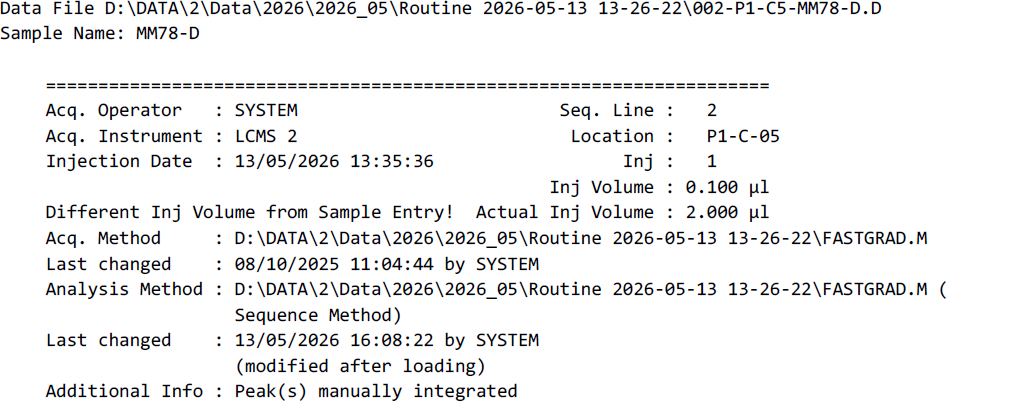


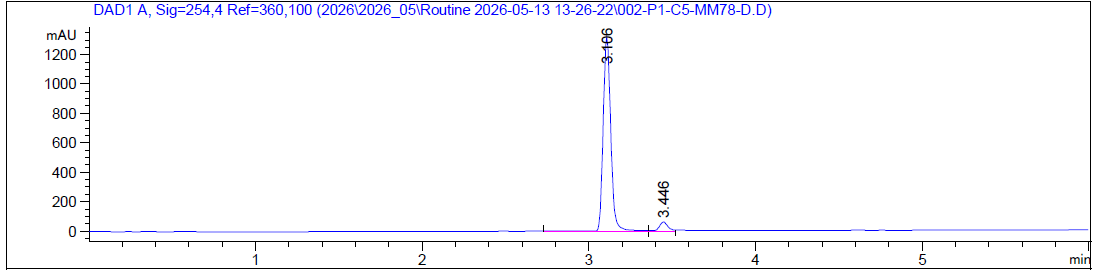


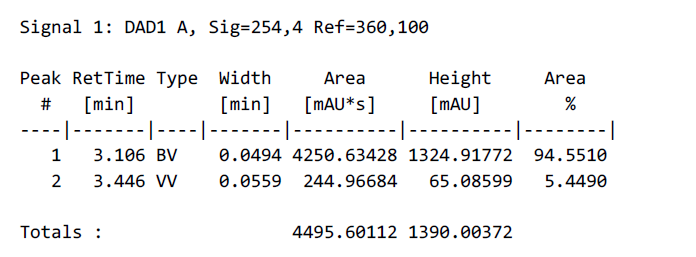


D-1-4d HRMS

D-1-4e purity

D-1-4e HRMS

D-2-5a purity

D-2-5a HRMS

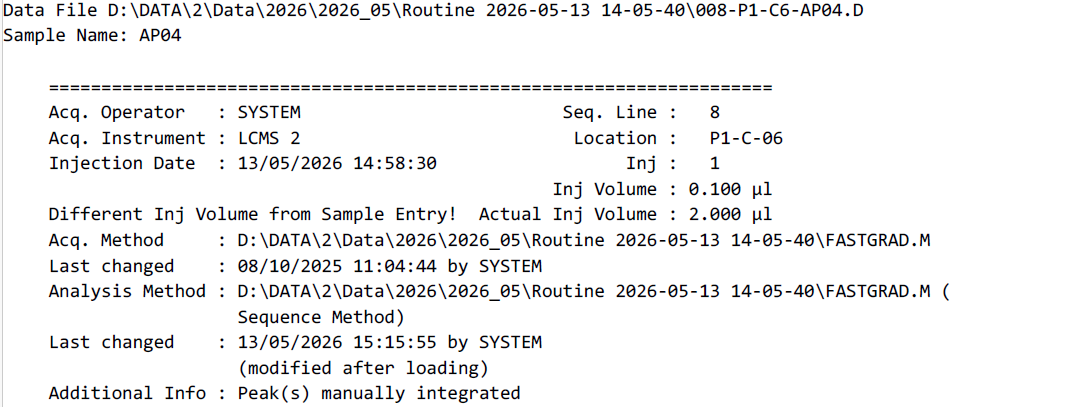
D-2-5b purity


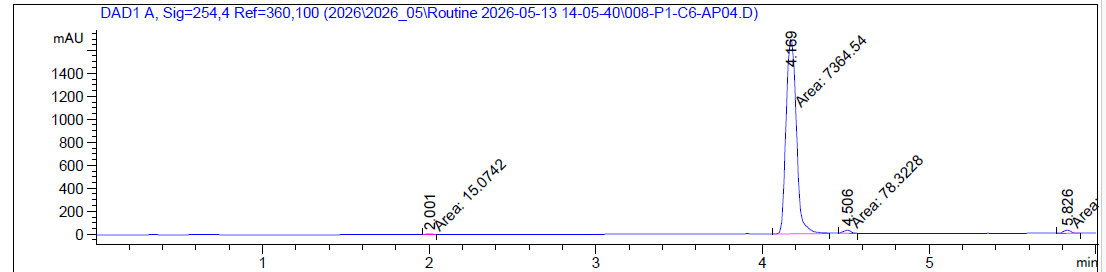


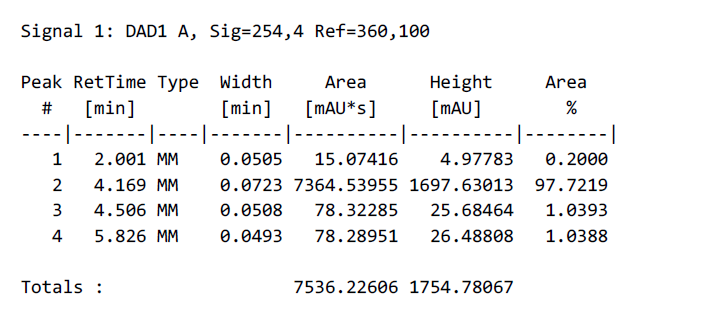


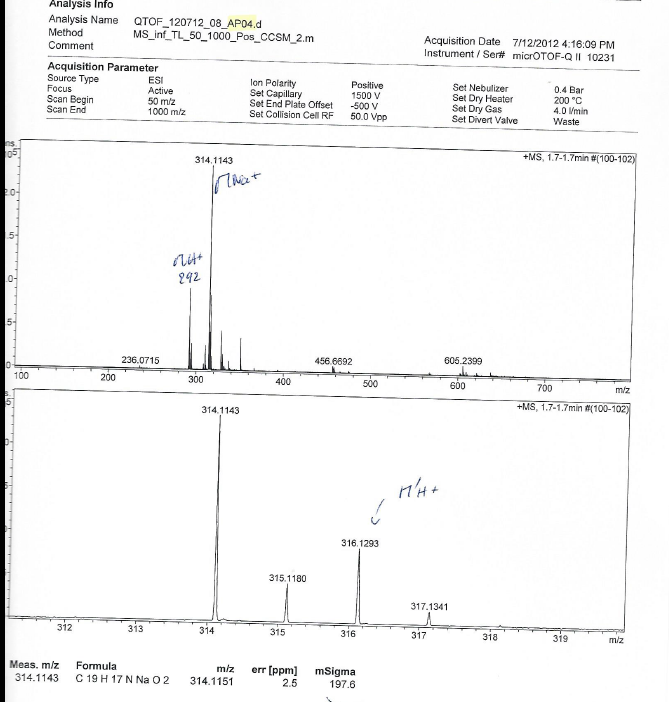
D-2-5b HRMS

D-3-6a purity

D-3-6a HRMS

D-3-6b purity


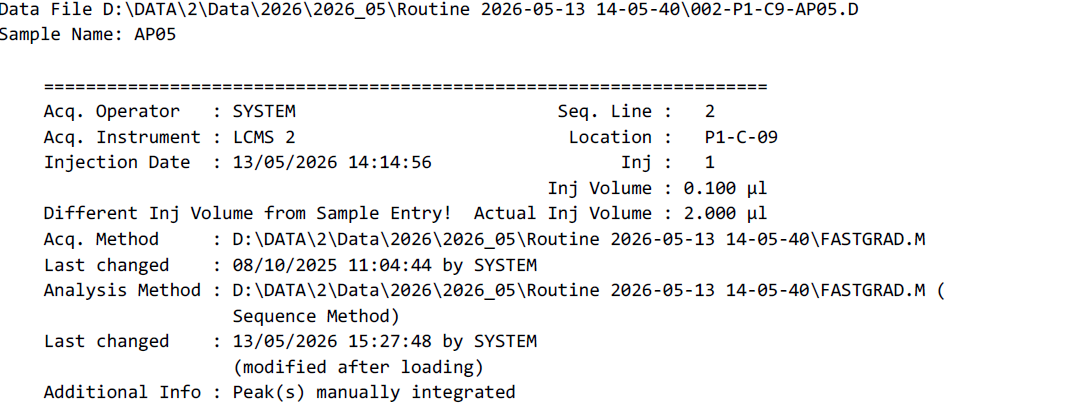


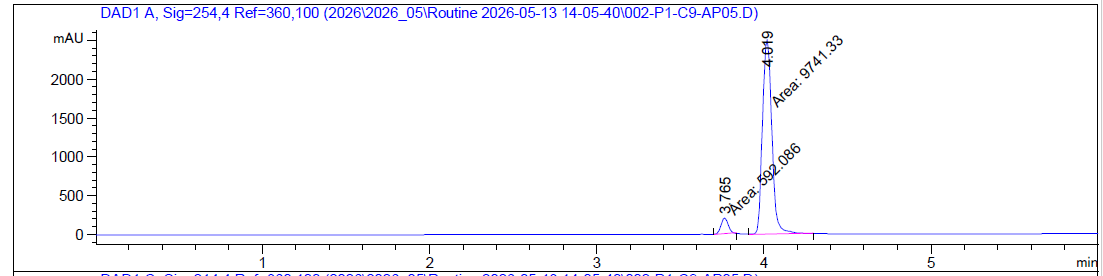


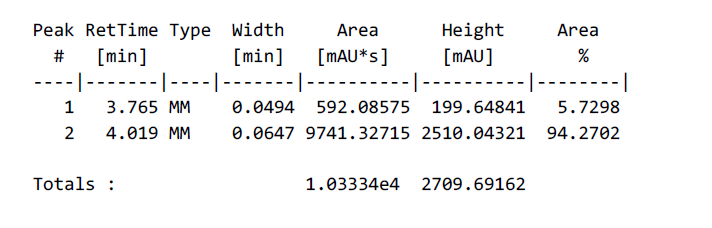


D-3-6b HRMS
