# Supplementary material for: Targeting Human Protein Kinase CK2 by a Library of Indeno[1,2‐b]Indoles: Contribution of Thermal Shift Assay to Pre‐Screening and Co‐Crystallization to Post‐Screening
Source: Arch Pharm (Weinheim). 2026 Aug 3;359(8):e70312. doi: 10.1002/ardp.70312 (PMC13430583; doi:10.1002/ardp.70312)
Supplement: Supplementary file 2 — Supporting File 2 [file ARDP-359-e70312-s001.doc]

**Supplemental Material: Novel Compounds and Biological Screening Results**

**Targeting Human Protein Kinase CK2 by a Library of Indeno[1,2-*b*]indoles: Contribution of Thermal Shift Assay to Pre-screening and Co-crystallization to Post-screening**

Matheus M. Guimarães^1,2^, Christian Werner^3^, Belen Leroy^1^, Johana Charles^1^, Jean Guillon^4^, Noël Pinaud^5^, Angélique Mularoni^1^, Marc Jean-Baptiste^1^, Perrine Ximenes^1^, Alexander Gast^6^, Helge Prinz^6^, Dagmar Aichele^6^, Alan G. Gonçalves^2^, Christelle Marminon^1^, Zouhair Bouaziz^7^, Joachim Jose^6^, Jean-Guy Delcros^1^, Karsten Niefind^3^, Marc Le Borgne^1^,*

1 Gastroenterology and technologies for health Team, Centre de Recherche en Cancérologie de Lyon, Centre Léon Bérard, CNRS 5286, INSERM 1052, Université Claude Bernard Lyon 1, Univ. Lyon, Lyon, France

2 Laboratory of Synthesis of Heterocycles and Glycoconjugates, Pharmaceutical Sciences Post-Graduation Program, Federal University of Paraná, Curitiba, PR, Brazil

3 Institute of Biochemistry, Department of Chemistry and Biochemistry, University of Cologne, Koln, Germany

4 INSERM, CNRS, ARNA, U1212, UMR 5320, UFR des Sciences Pharmaceutiques, Univ. Bordeaux, Bordeaux, France

5 ISM-CNRS UMR 5255, Univ. Bordeaux, Talence, France

6 Institute of Pharmaceutical and Medicinal Chemistry, PharmaCampus, University of Münster, Münster, Germany

7 Institut des Sciences Pharmaceutiques et Biologiques (ISPB), Faculté de Pharmacie, Université Claude Bernard Lyon 1, Univ. Lyon, Lyon, France

*Correspondence:

Prof. Dr., Marc Le Borgne, Gastroenterology and technologies for health, Cancer Research Center of Lyon, Faculty of Pharmacy-ISPB, 8 avenue Rockefeller, F-69373 Lyon Cedex 8, France

Email: marc.le-borgne@univ-lyon1.fr

| **Compound No.** | **InChI** | **Biological Activity**  **TSA^a^ using CK2α** | |
| --- | --- | --- | --- |
|  |  | Tm (°C) | ΔTm (°C) |
| **D-0-1B10** | 1S/C18H19NO4/c1-10(2)19-13-8-5-9-14(20)15(13)17(22)16(21)11-6-3-4-7-12(11)18(17,19)23/h3-4,6-7,10,22-23H,5,8-9H2,1-2H3 | 43.74 ± 0.68 | -0.43 |
| **D-0-3a** | 1S/C18H17NO4/c1-2-10-19-13-8-5-9-14(20)15(13)17(22)16(21)11-6-3-4-7-12(11)18(17,19)23/h2-4,6-7,22-23H,1,5,8-10H2 | 44.16 ± 0.14 | -0.01 |
| **D-0-3b** | 1S/C19H21NO4/c1-11(2)10-20-14-8-5-9-15(21)16(14)18(23)17(22)12-6-3-4-7-13(12)19(18,20)24/h3-4,6-7,11,23-24H,5,8-10H2,1-2H3 | 43.91 ± 0.63 | -0.26 |
| **D-0-1D1** | 1S/C24H23NO4/c1-15-13-19-21(20(26)14-15)23(28)22(27)17-9-5-6-10-18(17)24(23,29)25(19)12-11-16-7-3-2-4-8-16/h2-10,15,28-29H,11-14H2,1H3 | 44.03 ± 0.34 | -0.14 |
| **D-1-1A1** | 1S/C18H17NO2/c1-10(2)19-13-8-5-9-14(20)15(13)16-17(19)11-6-3-4-7-12(11)18(16)21/h3-4,6-7,10H,5,8-9H2,1-2H3 | 50.28 ± 0.18 | 6.11 |
| **D-1-4a** | 1S/C18H15NO2/c1-2-10-19-13-8-5-9-14(20)15(13)16-17(19)11-6-3-4-7-12(11)18(16)21/h2-4,6-7H,1,5,8-10H2 | 46.60 ± 0.54 | 2.44 |
| **D-1-4b** | 1S/C19H19NO2/c1-11(2)10-20-14-8-5-9-15(21)16(14)17-18(20)12-6-3-4-7-13(12)19(17)22/h3-4,6-7,11H,5,8-10H2,1-2H3 | 47.72 ± 0.18 | 3.55 |
| **D-1-1E6** | 1S/C24H21NO2/c1-15-13-19-21(20(26)14-15)22-23(17-9-5-6-10-18(17)24(22)27)25(19)12-11-16-7-3-2-4-8-16/h2-10,15H,11-14H2,1H3 | 44.71 ± 0.19 | 0.54 |
| **D-1-1A2** | 1S/C18H18N2O2/c1-9(2)20-12-7-4-8-13(21)15(12)16-17(20)10-5-3-6-11(19)14(10)18(16)22/h3,5-6,9H,4,7-8,19H2,1-2H3 | 51.09 ± 0.28 | 6.92 |
| **D-1-1B9** | 1S/C18H17NO3/c1-9(2)19-11-6-4-8-13(21)15(11)16-17(19)14-10(18(16)22)5-3-7-12(14)20/h3,5,7,9,20H,4,6,8H2,1-2H3 | 50.35 ± 0.57 | 6.18 |
| **D-1-1A4** | 1S/C23H25NO3/c1-13(2)11-12-27-18-10-5-7-15-19(18)23(26)21-20-16(8-6-9-17(20)25)24(14(3)4)22(15)21/h5,7,10-11,14H,6,8-9,12H2,1-4H3 | 43.43 ± 0.68 | -0.74 |
| **D-1-1A5** | 1S/C23H25NO3/c1-13(2)11-12-27-18-10-5-7-15-19(18)22-21(23(15)26)20-16(24(22)14(3)4)8-6-9-17(20)25/h5,7,10-11,14H,6,8-9,12H2,1-4H3 | 50.13 ± 0.77 | 5.96 |
| **D-1-1A6** | 1S/C19H19NO2/c1-10(2)20-14-8-11(3)9-15(21)16(14)17-18(20)12-6-4-5-7-13(12)19(17)22/h4-7,10-11H,8-9H2,1-3H3 | 48.85 ± 0.30 | 4.68 |
| **D-1-1A8** | 1S/C19H19NO2/c1-10(2)20-14-9-8-11(3)18(21)15(14)16-17(20)12-6-4-5-7-13(12)19(16)22/h4-7,10-11H,8-9H2,1-3H3 | 45.69 ± 0.50 | 1.52 |
| **D-1-1B1** | 1S/C20H21NO3/c1-10(2)21-13-8-11(3)9-14(22)17(13)18-19(21)16-12(20(18)23)6-5-7-15(16)24-4/h5-7,10-11H,8-9H2,1-4H3 | 50.53 ± 0.69 | 6.36 |
| **D-1-1B2** | 1S/C21H23NO3/c1-5-12-9-14-18(15(23)10-12)19-20(22(14)11(2)3)17-13(21(19)24)7-6-8-16(17)25-4/h6-8,11-12H,5,9-10H2,1-4H3 | 49.41 ± 0.31 | 5.24 |
| **D-1-4e** | 1S/C24H27NO3/c1-13(2)9-10-28-19-8-6-7-16-20(19)23-22(24(16)27)21-17(25(23)14(3)4)11-15(5)12-18(21)26/h6-9,14-15H,10-12H2,1-5H3 | 50.00 ± 0.98 | 5.83 |
| **D-2-2A1** | 1S/C18H15NO2/c1-10(2)19-13-8-5-9-14(20)15(13)16-17(19)11-6-3-4-7-12(11)18(16)21/h3-10,20H,1-2H3 | 46.00 ± 0.82 | 1.83 |
| **D-2-5a** | 1S/C18H13NO2/c1-2-10-19-13-8-5-9-14(20)15(13)16-17(19)11-6-3-4-7-12(11)18(16)21/h2-9,20H,1,10H2 | 44.77 ± 0.96 | 0.60 |
| **D-2-5b** | 1S/C19H17NO2/c1-11(2)10-20-14-8-5-9-15(21)16(14)17-18(20)12-6-3-4-7-13(12)19(17)22/h3-9,11,21H,10H2,1-2H3 | 45.29 ± 0.97 | 1.12 |
| **D-2-1F3** | 1S/C24H19NO2/c1-15-13-19-21(20(26)14-15)22-23(17-9-5-6-10-18(17)24(22)27)25(19)12-11-16-7-3-2-4-8-16/h2-10,13-14,26H,11-12H2,1H3 | 45.96 ± 0.73 | 1.79 |
| **D-3-1B4** | 1S/C18H13NO3/c1-9(2)19-16-10-5-3-4-6-11(10)18(22)15(16)14-12(20)7-8-13(21)17(14)19/h3-9H,1-2H3 | 46.52 ± 0.18 | 2.35 |
| **D-3-6a** | 1S/C18H11NO3/c1-2-9-19-16-10-5-3-4-6-11(10)18(22)15(16)14-12(20)7-8-13(21)17(14)19/h2-8H,1,9H2 | 48.56 ± 0.16 | 4.39 |
| **D-3-6b** | 1S/C19H15NO3/c1-10(2)9-20-17-11-5-3-4-6-12(11)19(23)16(17)15-13(21)7-8-14(22)18(15)20/h3-8,10H,9H2,1-2H3 | 46.24 ± 0.44 | 2.07 |
| **D-3-1D2** | 1S/C24H17NO3/c1-14-13-18(26)19-20-21(16-9-5-6-10-17(16)24(20)28)25(22(19)23(14)27)12-11-15-7-3-2-4-8-15/h2-10,13H,11-12H2,1H3 | 43.48 ± 0.67 | -0.69 |
| **CX-4945** | 1S/C19H12ClN3O2/c20-12-2-1-3-13(9-12)22-18-15-6-7-21-10-16(15)14-5-4-11(19(24)25)8-17(14)23-18/h1-10H,(H,22,23)(H,24,25) | 56.74 ± 0.24 | 12.57 |
| **SGC-CK2-1** | 1S/C20H21N7O/c1-3-19(28)25-16-8-15(5-4-12(16)2)23-17-9-18(24-14-6-7-14)27-20(26-17)13(10-21)11-22-27/h4-5,8-9,11,14,24H,3,6-7H2,1-2H3,(H,23,26)(H,25,28) | 55.09 ± 0.24 | 10.92 |
| **KDX1381** | 1S/C32H32F2N6O4S/c1-20(41)32-31(25-17-23(33)7-10-27(25)36-32)28-19-40(38-37-28)18-21-11-14-39(15-12-21)16-13-35-45(43,44)24-8-5-22(6-9-24)30-26(34)3-2-4-29(30)42/h2-10,17,19,21,35-36,42H,11-16,18H2,1H3 | 51.23 ± 0.31 | 7.06 |
| **CCh507** | 1S/C23H27N3O4S/c27-23(21-16-24-22-9-5-4-8-20(21)22)30-17-18-10-13-26(14-11-18)15-12-25-31(28,29)19-6-2-1-3-7-19/h1-9,16,18,24-25H,10-15,17H2 | 45.13 ± 0.32 | 0.96 |

^a^ The thermal shift assay was performed on a LightCycler 480 Real-Time PCR System (Roche) in 96-well white plates (Armadillo plate, Thermo Scientific) using an integration time of 120 ms. Each well contained 10 μL of PBS-0.9% glycerol-5% DMSO containing 5 μg CK2α, purified as described previously (1,2), 2.5× SYPRO Orange (Life Technologies) and 10 µM ligand (indeno[1,2-*b*]indole derivative, CX-4945, SGC-CK2-1, KDX1381 or CCh507). All assays were carried out in triplicate. Each plate was sealed with an optically clear foil and centrifuged for 1 min at 300 rpm before performing the assay. The plates were heated from 20 to 80 °C at a heating rate 0.01 °C/s. The fluorescence intensity was recorded at a rate of 50 acquisitions per °C with excitation at 483 nm and emission at 568 nm.

For each molecule, the experiment was done in triplicate. The ΔTm is calculated from the DMSO blank having a Tm value equal to 44.17 ± 0.42 °C.

Melting temperatures (Tm, °C) were determined using the TSA-CRAFT software that enables automatic analysis of TSA data exported from the Roche Lightcycler 480 software (3).

References:

[1] J. K. Hériché, F. Lebrin, T. Rabilloud, D. Leroy, and E. M. Chambaz, Y. Goldberg, “Regulation of protein phosphatase 2A by direct interaction with casein kinase 2alpha,” *Science* 276, no. 5314 (1997): 952–955, https://doi.org/10.1126/science.276.5314.952.

[2] L. Chantalat, D. Leroy, O. Filhol, et al., “Crystal structure of the human protein kinase CK2 regulatory subunit reveals its zinc finger-mediated dimerization,” *European Molecular Biology Organization Journal* 18, no. 11 (1999): 2930–2940, https://doi.org/10.1093/emboj/18.11.2930.

[3] P. H. Lee, X. X. Huang, B. T. Teh, and L. M. Ng, “TSA-CRAFT: A Free Software for Automatic and Robust Thermal Shift Assay Data Analysis,” *SLAS Discovery* 24, no. 5 (2019): 606–612, https://doi.org/10.1177/2472555218823547.
